# Second and third trimester fetal ultrasound population screening for risks of preterm birth and small-size and large-size for gestational age at birth: a population-based prospective cohort study: Fetal ultrasound screening for common adverse birth outcomes

**DOI:** 10.1186/s12916-020-01540-x

**Published:** 2020-04-07

**Authors:** Jan S. Erkamp, Ellis Voerman, Eric A. P. Steegers, Annemarie G. M. G. J. Mulders, Irwin K. M. Reiss, Liesbeth Duijts, Vincent W. V. Jaddoe, Romy Gaillard

**Affiliations:** 1 The Generation R Study Group, Erasmus MC, University Medical Center Rotterdam, P.O. Box 2040, 3000 CA Rotterdam, the Netherlands; 2 Department of Paediatrics, Erasmus MC, University Medical Center Rotterdam, Rotterdam, The Netherlands; 3 Department of Obstetrics and Gynaecology, Erasmus MC, University Medical Center Rotterdam, Rotterdam, The Netherlands; 4 Department of Paediatrics, Division of Neonatology, Erasmus MC, University Medical Center Rotterdam, Rotterdam, The Netherlands; 5 Department of Paediatrics, Division of Respiratory Medicine and Allergology, Erasmus MC, University Medical Center Rotterdam, Rotterdam, The Netherlands

**Keywords:** Fetal growth, Preterm birth, Small size for gestational age, Large size for gestational age, Cohort study, Screening, Third trimester ultrasound

## Abstract

**Background:**

Preterm birth, small size for gestational age (SGA) and large size for gestational age (LGA) at birth are major risk factors for neonatal and long-term morbidity and mortality. It is unclear which periods of pregnancy are optimal for ultrasound screening to identify fetuses at risk of preterm birth, SGA or LGA at birth. We aimed to examine whether single or combined second and third trimester ultrasound in addition to maternal characteristics at the start of pregnancy are optimal to detect fetuses at risk for preterm birth, SGA and LGA.

**Methods:**

In a prospective population-based cohort among 7677 pregnant women, we measured second and third trimester estimated fetal weight (EFW), and uterine artery pulsatility and umbilical artery resistance indices as placenta flow measures. Screen positive was considered as EFW or placenta flow measure < 10th or > 90th percentile. Information about maternal age, body mass index, ethnicity, parity, smoking, fetal sex and birth outcomes was available from questionnaires and medical records. Screening performance was assessed via receiver operating characteristic (ROC) curves and area under the curve (AUC) along with sensitivity at different false-positive rates.

**Results:**

Maternal characteristics only and in combination with second trimester EFW had a moderate performance for screening for each adverse birth outcome. Screening performance improved by adding third trimester EFW to the maternal characteristics (AUCs for preterm birth 0.64 (95%CI 0.61 to 0.67); SGA 0.79 (95%CI 0.78 to 0.81); LGA 0.76 (95%CI 0.75; 0.78)). Adding third trimester placenta measures to this model improved only screening for risk of preterm birth (AUC 0.72 (95%CI 0.66 to 0.77) with sensitivity 37% at specificity 90%) and SGA (AUC 0.83 (95%CI 0.81 to 0.86) with sensitivity 55% at specificity 90%). Combining second and third trimester fetal and placental ultrasound did not lead to a better performance as compared to using only third trimester results.

**Conclusions:**

Combining single third trimester fetal and placental ultrasound results with maternal characteristics has the best screening performance for risks of preterm birth, SGA and LGA. As compared to second trimester screening, third trimester screening may double the detection of fetuses at risk of common adverse birth outcomes.

**Electronic supplementary material:**

The online version of this article (10.1186/s12916-020-01540-x) contains supplementary material, which is available to authorized users.

## Background

Preterm birth, small size for gestational age (SGA) and large size for gestational age (LGA) at birth explain up to 30% of neonatal death and are strong risk factors for short-term and long-term morbidity [1, 2]. The majority of newborns who experience abnormal fetal growth are unidentified until birth [3–6]. SGA or LGA newborns who have not been identified antenatally have strongly increased risks of morbidity and mortality, compared to those who have been identified antenatally [6–9]. Abnormal fetal growth is an important reason for induction of labour and is therefore a common cause of induced preterm birth [3, 10]. However, studies have shown that spontaneous preterm birth is often preceded by impaired or accelerated fetal growth [3, 9]. Current pregnancy care protocols include dating ultrasounds and detailed structural ultrasounds at 20 weeks gestational age (GA) to assess congenital anomalies and fetal size [11, 12]. Third trimester ultrasound screening is mostly used in selected populations. Technological developments in obstetric ultrasounds may lead to future changes in ultrasound screening protocols, such as early-pregnancy size and congenital anomaly assessment and third trimester growth assessment. The performance of routine third trimester ultrasound screening, independent of other maternal and fetal characteristics, is not clear. A review of eight controlled trials did not suggest consistent benefits of ultrasound after 24 weeks GA on pregnancy outcomes [13]. A prospective observational cohort study among 3977 nulliparous women suggested that third trimester ultrasound, in addition to second trimester ultrasound, tripled the detection of fetuses subsequently born SGA compared to selective third trimester ultrasound [14].

We used data from a population-based observational study among 7670 pregnant women to examine whether single or combined second or third trimester fetal and placental ultrasound examinations, in addition to maternal characteristics, are optimal to detect fetuses at risk for preterm birth, SGA and LGA.

## Methods

### Study design

This study was embedded in the Generation R Study, a population-based prospective cohort study from early pregnancy onwards in Rotterdam, the Netherlands [15]. The study has been approved by the local Medical Ethical Committee (MEC 198.782/2001/31). Written consent was obtained from all women. All pregnant women were enrolled between 2001 and 2005. The response rate at birth was 61%, which was calculated by dividing the number of participating live born children by the total number of live born children born in the study area during the inclusion period [16]. A total of 8879 women were enrolled during pregnancy. We excluded women without second and third trimester ultrasound data (*n* = 1130), non-singleton live-births (*n* = 33), and women with outcome data missing (*n* = 46). This led to a population for analysis of 7670 pregnant women (Additional file 1, Figure S1 shows the flowchart for the population for analysis). Additional file 2 contains a Strengthening the Reporting of Observational Studies in Epidemiology (STROBE) statement for the current study [17].

### Maternal characteristics at the start of pregnancy

We selected maternal characteristics known at the start of pregnancy, which are important determinants of adverse birth outcomes [3, 18–20]. Maternal age was assessed at enrolment and categorized; < 25.0 years, 25.0–34.9 years, ≥ 35.0 years [3]. Maternal height (cm) and weight (kg) were measured without shoes and heavy clothing at enrolment and BMI (kg/m^2^) was calculated and categorized for clinical purposes: normal weight (BMI < 25 kg/m^2^), overweight (BMI 25.0–30.0 kg/m^2^) and obese (BMI ≥ 30.0 kg/m^2^) [19]. Information about ethnicity and parity and smoking was obtained by questionnaire and categorized as previously described [3, 18].

### Second and third trimester fetal and placental ultrasounds

Ultrasound examinations were carried out in two dedicated research centres in first (median 13.2 weeks GA, interquartile range (IQR) 12.2 to 14.7), second (median 20.5 weeks GA, IQR 19.9 to 21.3) and third trimester (median 30.4 weeks GA, IQR 29.8 to 30.9) [3]. We established GA by using data from the first ultrasound [3]. In second and third trimesters, we measured fetal head circumference, abdominal circumference (AC) and femur length to the nearest millimeter using standardized procedures [21]. Estimated fetal weight (EFW) was calculated using the formula of Hadlock et al., in line with clinical practice [22]. GA-adjusted SDS for growth measures were based on reference growth charts from the whole study population [3]. In line with clinical practice, we defined screen-positive as EFW or AC in the lowest or highest decile in second or third trimester [5, 8, 14, 23, 24]. Both extremes of EFW and AC are associated with a higher risk of common adverse birth outcomes and perinatal morbidity and mortality [3, 14]. This approach leads to one screening test for all adverse birth outcomes, which strongly improves ease-of-use in clinical practice. However, EFW > 90th percentile is not associated with an increased risk of delivering a SGA newborn. Similarly, EFW < 10th percentile is not associated with an increased risk of delivering a LGA newborn. Thus, defining screen positive as EFW < 10th percentile and > 90th percentile for all adverse birth outcomes in our screening models may reduce the observed screening performance. The performance of the screening model may be improved when we define screen positive separately for SGA (as EFW < 10th percentile) and for LGA (as EFW > 90th percentile). We consider one combined screening test for all adverse birth outcomes more applicable for clinical practice, but to assess whether this affects the observed screening performance, we also evaluated screening performance of models in which we defined “screen-positive” separately for SGA (EFW < p10) and LGA (EFW > p90). Second-to-third trimester EFW or AC change was classified screen-positive if the change was in the lowest or highest decile.

Uterine artery resistance indices (UtA-RI) and umbilical artery pulsatility indices (UA-PI) are measures of vascular resistance in the uterine and umbilical arteries, respectively. Increased UtA-RI and UA-PI are associated with impaired placental vascular development and increased risks of abnormal intrauterine growth and adverse perinatal outcomes [23, 25–29]. These parameters may therefore be of additional value in clinical screening models. These parameters were derived from flow velocity waveforms in second and third trimesters [30]. We defined screen-positive UtA-RI or UA-PI or second-to-third trimester change as a value in the highest decile.

### Birth outcomes

Information about offspring sex, GA and weight at birth, gestational hypertensive disorders, assisted vaginal delivery and cesarean delivery was obtained from medical records [15]. GA-adjusted SDS for birth weight was constructed using North European growth standards [31]. Preterm birth was defined as GA < 37 weeks at birth. Spontaneous preterm birth was defined as spontaneous preterm labour or preterm premature rupture of membranes resulting in birth < 37 weeks’ GA. According to clinical standards, SGA and LGA at birth were defined as a GA-adjusted birth weight < 10th and > 90th percentile in the study cohort, respectively.

### Statistical analyses

First, we calculated the absolute percentages of screen positive second and third trimester fetal ultrasounds among newborns born preterm, SGA and LGA. Second, we aimed to assess screening performance for preterm birth, SGA and LGA based on different predefined screening models. We constructed five predefined logistic regression models for screening of preterm birth, SGA and LGA, respectively. Preterm birth, SGA and LGA were the dependent variables in these different predefined logistic regression models. For each logistic regression model, we assessed the variance explained of the model. We obtained predicted values from these regression models and further assessed model performance via receiver operating characteristic (ROC) curves and calculation of the area under the curve (AUC), along with the sensitivity at different false-positive rates (1-specificity). The five predefined logistic regression models for screening of preterm birth, SGA and LGA were as follows: (1) maternal characteristics model including maternal age, BMI, ethnicity, parity and smoking and fetal sex; (2) second trimester model (model 1 plus screening result based on second trimester EFW); (3) third trimester model (model 1 plus screening result based on third trimester EFW); (4) combined second and third trimester model (model 1 plus screening result based on second and third trimester EFW); (5) second-to-third trimester fetal growth model (model 4 plus second-to-third trimester EFW change). To compare model performance of the different predefined models, we assessed the change in effect size of the obtained AUCs from the different models. If the change in effect size was considered clinically relevant, we used the method by DeLong et al. for assessing whether the AUCs for two or more correlated receiver operating characteristic curves were statistically significantly different [32]. Positive and negative predictive values (PPV, NPV) and positive and negative likelihood ratios (PLR, NLR) at a 10% false-positive rate (90% specificity) were calculated for our best model. Third, in a subsample of women with placenta flow measures available, we assessed the additional screening performance of placenta measures by adding second and third trimester UA-PI and UtA-RI screening results to the five models using a similar approach. To test the robustness of our findings, we performed 8 formal sensitivity analyses. We assessed (1) whether screening performance for spontaneous preterm birth was similar to screening performance for any preterm birth, (2) whether using stricter cut-off values to define screen-positive results improved screening performance (EFW < 5th percentile or EFW > 95th percentile), (3) whether our models improved when we used AC instead of EFW, (4) whether using only UA-PI or UtA-RI screening results leads to comparable screening performance as using both measurements combined, (6) whether defining “screen-positive” for individual outcomes separately (screen positive as EFW < 10th percentile only for SGA and screen positive as EFW > 90th percentile only for LGA), instead of defining screen-positive as either EFW < 10th or EFW > 90th for all adverse birth outcomes, affects screening performance, (7) whether the screening performance changed when the outcome SGA was defined as moderate or extreme SGA (gestational-age-adjusted birth weight < 5th or < 3rd percentile, respectively) or defined as moderate or extreme LGA (gestational-age-adjusted birth weight > 95th or > 97th percentile, respectively), (8) whether performance of our model was similar for selecting SGA or LGA newborns with adverse outcomes (SGA pregnancies complicated by gestational hypertensive disorders and LGA pregnancies resulting in delivery using assisted vaginal delivery or cesarean section). Finally, to assess how maternal characteristics affect our obtained screening performance of the different screening models, we assessed the screening performance of second and third trimester ultrasound without incorporating maternal characteristics in the models. To deal with missing values, we added a missing category for each maternal and fetal characteristic to the models. This approach resembles clinical practice. Analyses were performed using the Statistical Package of Social Sciences version 24.0 for Windows (IBM Corp., Armonk, NY, USA).

## Results

### Participants characteristics

Table 1 shows that 345 (4.5%) newborns were born preterm, 768 (10%) were SGA, and 767 (10%) were LGA at birth. Additional file 1, Table S1 gives all fetal and placental characteristics. Non-response analyses showed that women without placental measurements were more likely to have a higher BMI and lower educational level (Additional file 1, Table S2). Of all newborns with a second trimester EFW < 10th percentile or > 90th percentile, 91 (5.9%) were born preterm, 214 (13.9%) were born SGA and 179 (11.7%) were born LGA. Of all newborns with a third trimester EFW < 10th or > 90th percentile, 110 (7.2%) were born preterm, 335 (21.8%) were born SGA and 277 (18.1%) were born LGA (Table 2). In univariate logistic regression analyses, all maternal exposures were associated with at least one of the adverse birth outcomes, whereas EFW was associated with all three adverse birth outcomes (results available upon request).

**Table 1:** Characteristics of mothers and their children (*N* = 7670)

Characteristics	Value^a^
Maternal characteristics	
Age, median (IQR), years	30.3(25.9 to 33.4)
< 25, no. (%)	1573(20.5)
25–35, no. (%)	4972(64.8)
> 35, no. (%)	1125(14.7)
Height, mean (SD) (cm)	167.3(7.4)
Weight, mean (SD) (kg)	69.3(13.2)
Body mass index^1^, mean (SD) (kg/m^2^)	24.8(4.5)
Normal, no. (%)	4709(61.8)
Overweight, no. (%)	1979(26.0)
Obese, no. (%)	932(12.2)
Education, no. higher education (%)	3055(42.9)
Race/ethnicity, no. (%)	
Dutch or European, no. (%)	4289(58.2)
Surinamese, no. (%)	655(8.9)
Turkish, no. (%)	673(9.1)
Moroccan, no. (%)	473(6.4)
Cape Verdian or Dutch Antilles, no. (%)	560(7.6)
Parity, no. nulliparous (%)	4308(56.6)
Smoking, no. (%)	
None, no. (%)	4967(72.8)
Early pregnancy only, no. (%)	595(8.7)
Continued, no. (%)	1261(18.5)
Birth characteristics	
Males, no. (%)	3861(50.3%)
Gestational age, median (IQR), weeks	40.1(39.1 to 41.0)
Birth weight, mean (SD) grams	3423(544)
Preterm birth^2^, no. (%)	345(4.5)
Spontaneous preterm birth, no. (%)	294(3.0)
Small-size for gestational age^3^ < 10 birth centile (<− 1.4SDS), no. (%)	768(10)
Large-size for gestational age^3^ > 90 birth centile (> 1.18SDS), no. (%)	767(10)
Cesarean delivery, no. (%)	836(11.9)
Assisted vaginal delivery, no. (%)	964(13.8)
Apgar score below 7 at 5 min, no. (%)	78(1.0)

^a^Values are observed data and represent means (SD), medians (IQR) or number of subjects (valid %). *Abbreviations*: *IQR* inter quartile range, *SD* standard deviation

^1^Body mass index is defined as normal (BMI < 25), overweight (BMI 25–30), obese (BMI > 30)

^2^Preterm birth is defined as birth < 37 weeks of gestational age

^3^SGA is defined as < 10th percentile of gestational age- and sex-adjusted birth weight; LGA is defined as > 90th percentile of gestational age- and sex-adjusted birth weight

**Table 2:** Adverse birth outcomes by second and third trimester estimated fetal weight screening results (*N* = 7670)^a^

	Preterm birth			Small size for gestational age at birth			Large size for gestational age at birth		
	Yes	No	Total	Yes	No	Total	Yes	No	Total
2nd trimester									
Estimated fetal weight < 10th percentile (screen-positive)	41 (5.3%)	726 (94.7%)	767	192 (25.0%)	575 (75.0%)	767	30 (3.9%)	737 (96.1%)	767
Estimated fetal weight 10–90th percentile (screen negative)	254 (4.1%)	5882 (95.9%)	6136	554 (9.0%)	5582 (91.0%)	6136	588 (9.6%)	5548 (90.4%)	6136
Estimated fetal weight > 90th percentile (screen-positive)	50 (6.5%)	717 (93.5)	767	22 (2.9%)	745 (97.1%)	767	149 (19.4%)	618 (80.6%)	767
*Total*	345	7325	7670	768	6902	7670	767	6903	7670
3rd trimester	Yes	No	Total	Yes	No	Total	Yes	No	Total
Estimated fetal weight < 10th percentile (screen-positive)	75 (9.8%)	692 (90.2%)	767	331 (43.2%)	436 (58%)	767	4 (0.5%)	763 (99.5%)	767
Estimated fetal weight 10–90th percentile (screen negative)	235 (3.8%)	5901 (96.2%)	6136	433 (7.1%)	5703 (92.9%)	6136	490 (8%)	5646 (92%)	6136
Estimated fetal weight > 90th percentile (screen-positive)	35 (4.6%)	732 (95.4%)	767	4 (0.5%)	763 (99.5%)	767	273 (35.6%)	494 (64.4%)	767
*Total*	345	7325	7670	768	6902	7670	767	6903	7670

^a^Values are absolute numbers (% of total within the corresponding screening category)

### Screening for risks of preterm birth

**Fig. 1:**
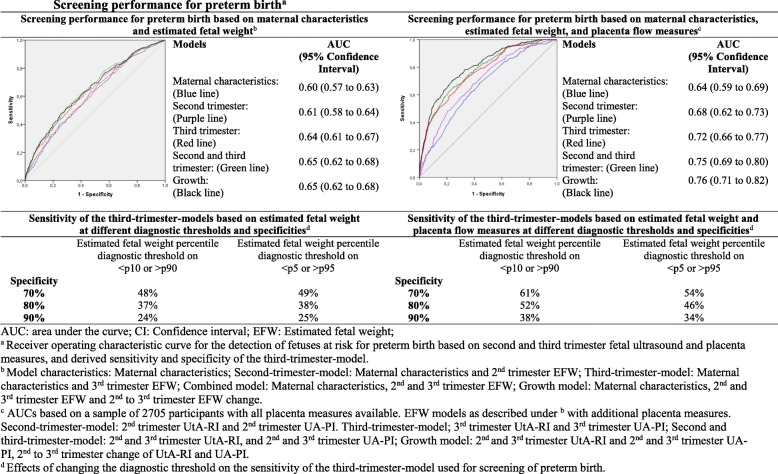
Screening performance for preterm birth

Figure 1 shows that the maternal characteristics model had a moderate performance for the detection of preterm birth (AUC 0.60 (95% CI 0.57 to 0.63), which did not improve by adding second trimester EFW (AUC 0.61 (95% CI 0.58 to 0.64)). Screening improved by adding third trimester EFW (AUC 0.64 (95% CI 0.61 to 0.67) to the maternal characteristics model (*p* value for AUC comparison to the maternal characteristics model < 0.01, Additional file 1, Table S3). AUC effect estimates did not further improve by combining second and third trimester EFW results or using EFW change. Adding placenta flow measures to the third trimester EFW model strongly improved detection of preterm birth (AUC of 0.72 (95% CI 0.66 to 0.77), *p* value for model comparison to the third trimester EFW model < 0.01, Additional file 1, Table S3). Compared to the second trimester model, the third trimester model with placenta flow measures nearly doubled detection of fetuses at risk of preterm birth, as sensitivity increases from 19% for the second trimester model to 38% for the third trimester model with placenta flow measures (PLR 3.8; NLR 0.69; PPV 15%; NPV: 97%) at 90% specificity (Fig. 1, Additional file 1, Table S4).

We observed similar model performances when we only took spontaneous preterm birth into account (Additional file 1, Figure S2). Using stricter diagnostic cut-offs led to similar AUCs and sensitivities (Fig. 1, Additional file 1, Figure S3). We did not observe differences in results when we used AC instead of EFW (Additional file 1, Figure S4). Overall, combined use of UtA-RI and UA-PI tended to be better than separate use (Additional file 1, Figure S5). Additional file 1, Figure S6 shows that without maternal characteristics, screening performance of the third trimester model with placenta flow measures for preterm birth was considerably lower.

### Screening for risks of small size and large size for gestational age at birth

**Fig. 2:**
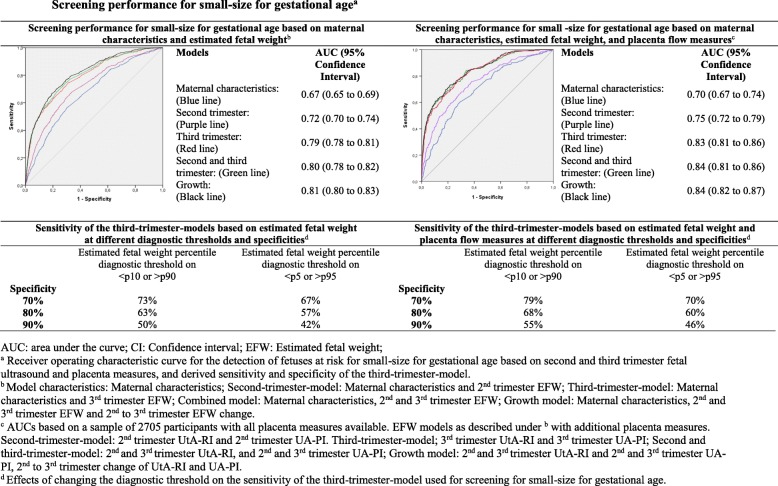
Screening performance for small size for gestational age

**Fig. 3:**
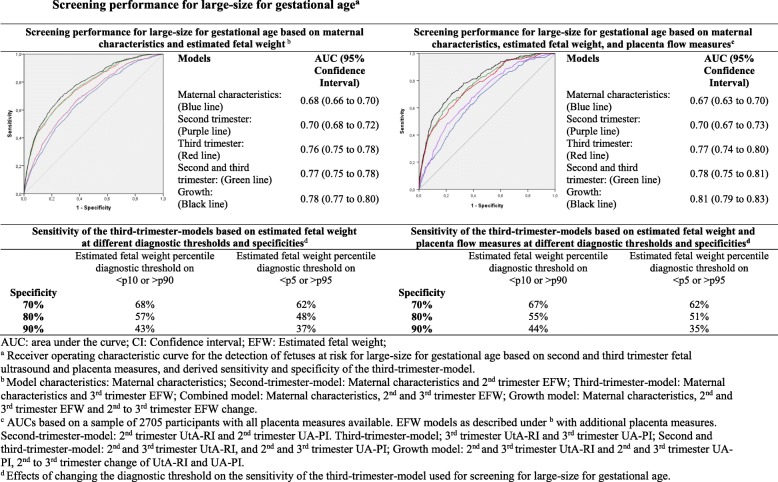
Screening performance for large size for gestational age

The maternal characteristics model and second trimester model had a moderate screening performance for detection of SGA at birth (AUCs 0.67 (95% CI 0.65 to 0.69) and 0.72 (95% CI 0.70 to 0.74), respectively) (Fig. 2). Compared to these models, the third trimester model significantly improved detection (AUC 0.79 (95% CI 0.78 to 0.81) with a sensitivity of 50% at 90% specificity (*p* value for AUC comparison to the maternal characteristics model and second trimester model < 0.01, Additional file 1, Table S3). Compared to the second trimester model, the third trimester model increased detection of fetuses at risk of SGA by a third, as sensitivity increases from 33% for the second trimester model to 50% for the third trimester model at 90% specificity (Fig. 2, Additional file 1, Table S4). Effect estimates of the AUCs did not further clinically improve by combining second and third trimester EFW results or using EFW change. Adding placenta flow measures to the third trimester model did slightly improve screening performance for SGA at birth (AUC 0.83 (95% CI 0.81 to 0.86) *p* value for AUC comparison to the third trimester model < 0.01, Fig. 2, Additional file 1, Table S3) leading to a sensitivity of 55% at 90% specificity (PLR 5.5; NLR 0.5; PPV 38%; NPV 95%). The third trimester model had the best screening performance for detecting LGA with an AUC of 0.76 (95% CI 0.75 to 0.78) and corresponding sensitivity of 43% at 90% specificity (Fig. 3). Compared to the second trimester model, the third trimester model increased the detection of fetuses at risk of LGA by a third, as the sensitivity increases from 28% for the second trimester model to 43% for the third trimester model (PLR 4.3; NLR 0.63; PPV 32%; NPV 93%) at 90% specificity (Fig. 3, Additional file 1, Table S4). Adding placenta flow measures to the third trimester model did not improve LGA screening performance.

Model performance was the same when screen-positive was defined separately for SGA and LGA, as when screen-positive was defined as one screening test for both SGA and LGA (Additional file 1, Figure S7). When we used stricter diagnostic cut-offs (screen-positive defined as EFW < 5th or > 95th percentile), the sensitivities for detection of SGA and LGA slightly decreased (Figs. 2 and 3 respectively, ROCs and AUCs in Additional file 1, Figure S3). When we used stricter outcome cut-offs (extreme SGA and LGA defined as gestational-age-adjusted birth weight < 3rd or > 97th percentile, and moderate SGA or LGA defined as gestational-age-adjusted birth weight < 5th or > 95th percentile, respectively), the model performance slightly improved as compared to our main analysis (sensitivities, ROCs and AUCs in Additional file 1, Figure S8 and Figure S9). When we assessed screening performance for SGA newborns with pregnancies complicated by gestational hypertensive disorders and LGA newborns with pregnancies resulting in assisted vaginal delivery or cesarean section, we observed similar model performance as our main analysis (Additional file 1, Figure S10). We did not observe differences in results when we used AC instead of EFW (Additional file 1, Figure S4). When we excluded maternal smoking from the models, results were similar (findings not shown). Without incorporating maternal characteristics in the screening models, screening performance of the third trimester model for SGA and LGA was considerably lower (Additional file 1, Figure S6).

## Discussion

Our results suggest that third trimester ultrasound examination in addition to maternal characteristics has the best screening performance for detecting fetuses at risk for preterm birth, SGA and LGA, compared to second trimester ultrasound or combined second and third trimester ultrasound. Compared to second trimester ultrasound screening, third trimester ultrasound screening would nearly double detection of fetuses at risk of these adverse birth outcomes in a low-risk population.

### Interpretation of main findings

Preterm birth, SGA and LGA are strongly related to perinatal morbidity and mortality and have long-term consequences for disease risk [2, 4, 33]. Abnormal fetal growth and impaired placental function are important risk factors for adverse birth outcomes, with the strongest associations observed for third trimester fetal and placental measures [3–5, 29]. Despite these observed associations, the additional clinical value for third trimester screening for fetuses at risk for common adverse birth outcomes remains unclear. A review of 13 controlled trials showed no beneficial effects of routinely performed ultrasound after 24 weeks GA on pregnancy outcomes [13]. These trials were mainly performed in the early 1990s. Recent developments in ultrasound techniques and treatment protocols, and changes in prevalence of women at risk of abnormal fetal growth limit the applicability of these results to current clinical practice. Technological ultrasound advancements in obstetrics may lead to implementation of fetal size and anomaly scans in first trimester and fetal growth assessment later in pregnancy. Further insight into the optimal period for ultrasound screening for adverse birth outcomes is therefore urgently needed.

Despite reported associations of suboptimal fetal growth and impaired placental function with preterm birth, no previous studies assessed the screening performance of second and third trimester ultrasound for preterm birth risk [3, 34]. We observed that third trimester fetal and placental ultrasound together with maternal characteristics had the best screening performance for preterm birth. We did not find a benefit of second to third trimester EFW change for screening for preterm birth, although previously published work from our cohort showed that second to third trimester EFW change was associated with the risk of preterm birth [3]. In this previous analysis, we only assessed the association of second to third trimester EFW change with the risk of preterm birth and did not consider second or third trimester fetal size in the analysis. Contrary, in our current analysis, we assessed the screening performance for preterm birth of the addition of second to third trimester EFW change to second and third trimester fetal size, and observed it did not further improve screening performance. Thus, it seems that an association between second to third trimester EFW change with the risk of preterm birth is present, but that this does not add to screening performance for preterm birth when we also consider second and third trimester fetal size*.* The additional value of placenta measures to the screening model may be explained by the role of placental dysfunction in preterm birth [1]. We observed the strongest screening performance for using a combination of umbilical and uterine artery resistance indices. However, differences compared to single use of either measurement were small. As the umbilical artery pulsatility indices are technically easier to measure, this measure might be most appropriate for use in clinical practice. Overall, in our relatively healthy low-risk population, the combination of third trimester fetal and placental ultrasound with maternal characteristics led to a doubling of antenatally identified newborns at risk for preterm birth compared to second trimester ultrasound or maternal characteristics only. A limited number of previous studies explored screening performance by single and combined second and third trimester EFW or AC measurements for prediction of SGA or LGA, taking into account maternal characteristics. A retrospective study among 3520 women reported a moderate screening performance for SGA with a sensitivity of 41.8% at 90% specificity using a combination of maternal factors, first trimester chemistry results and second trimester EFW and placenta measures [35]. Another retrospective cohort study among 1979 women reported that adding maternal characteristics and third trimester fetal and placental ultrasound to second trimester ultrasound results improved sensitivity from 51.3 to 69.7% for SGA and from 44.1 to 59.4% for LGA at 90% specificity [36]. A recent cohort study among 3440 pregnancies assessed the screening value of single versus serial fetal biometry at 28, 32 and 36 weeks GA for SGA and LGA [37]. This study observed that single fetal biometry at 32 weeks had a higher sensitivity than longitudinal analysis from more observations projecting EFW at 40 weeks [37]. In our study, the third trimester ultrasound was performed at an average of 30 weeks of gestation, as compared to an average 34 to 36 weeks of gestation in other studies [37, 38]. Although screening performance of third trimester ultrasound may improve when performed later in the third trimester, third trimester ultrasound screening around 30 weeks’ gestation is valuable as it offers a larger window for interventions. In our study, we observed that third trimester fetal and placental ultrasound together with maternal characteristics had the best screening performance for SGA and LGA. Already in our low-risk population, this approach led to a third increase in detection of fetuses at risk of SGA or LGA compared to second trimester ultrasound screening. We did not observe additional screening benefit for combining second and third trimester ultrasounds or for using AC instead of EFW.

It is well-established that newborns born SGA or LGA may be both constitutionally or pathologically small or large for their gestational age [39]. It has been suggested that newborns who are pathologically small or large for their gestational age due to abnormal fetal growth have increased risks of morbidity and mortality, as compared to those newborns who are constitutionally small or large for their gestational age [39]. To better distinguish potential pathological SGA and LGA newborns from constitutional SGA and LGA newborns, we also assessed the screening performance of our screening models for moderate and extreme SGA and LGA, and for SGA and LGA complicated by pregnancy or delivery complications. We found that the screening performance was similar. This suggests our third trimester screening model may aid in the identification of newborns who are pathologically small or large for their gestational age. We did not use customized birth weight centiles for classification of abnormal size at birth as a method to distinguish potential pathological SGA and LGA newborns from constitutional SGA or LGA newborns, as previous studies have not shown strong results regarding the use of customized charts to identify SGA or LGA newborns at higher risk of mortality and adverse short-term and long-term outcomes [40, 41]. A limitation of our cohort is that we do not have extensive information available on neonatal morbidity. Further studies are needed to replicate our findings and to assess whether our screening model identifies SGA and LGA born newborns at risk of morbidity and mortality, considering more detailed measures of neonatal morbidity.

Overall, we observed slightly lower sensitivities for screening for SGA and LGA than previous studies, which could be explained by taking into account maternal characteristics, our relatively healthy low-risk population and the earlier timing of third trimester ultrasound [37, 38]. As maternal characteristics are simple and cost-effective measurements, easily available within clinical practice, we specifically aimed to assess their screening performance for screening of adverse birth outcomes within low-risk populations and the subsequent additional screening performance of more expensive and time-consuming fetal and placental ultrasound measurements. We found that in absence of maternal characteristics, the screening models had an inferior screening performance compared to when maternal characteristics were taken into account but the third trimester fetal and placental ultrasound still had the best screening performance for adverse birth outcomes. Thus, our findings underline the importance of considering maternal characteristics within low-risk populations for screening of adverse birth outcomes and the potential value of third trimester ultrasound.

Our findings suggest that implementation of third trimester fetal and placental ultrasound, combined with common maternal characteristics, would nearly double detection of fetuses at risk for common adverse birth outcomes compared to second trimester ultrasound and provides further evidence for critical evaluation of current obstetric care guidelines. Improved detection of fetuses at risk of preterm birth, SGA and LGA provides the clinician the opportunity to optimize monitoring and interventions [42]. Monitoring could be intensified by additional assessments of fetal size, cervical length and umbilical artery waveforms using (Doppler) ultrasound and fetal wellbeing using cardiotocography, which might further improve detection of fetuses at risk of adverse outcomes whom may benefit from interventions, such as administering steroids for fetal lung maturation if preterm birth is imminent or termination of pregnancy if signs of placental insufficiency occur. Previous studies have shown that SGA or LGA newborns who were identified antenatally have lower risks of morbidity and mortality, compared to those who were unidentified antenatally [6–9]. However, it has also been suggested that prenatal diagnosis of abnormal fetal growth may lead to poorer outcomes due to subsequent interventions [43]. Benefits due to identification of true positives versus harm caused by false positives and interventions should be evaluated. Future well-designed randomized controlled trials are needed to confirm our results and to assess whether the advantages of screening outweigh the potential harm from parental anxiety and iatrogenic morbidity, in contemporary low-risk populations.

### Strengths and limitations

We had a prospective data collection from early pregnancy onwards and a large sample of 7670 women with fetal growth measurements available. The non-response at baseline might have led to selection of a more healthy population, which might affect the generalizability of results to high-risk populations. We also had a relatively small number of cases of adverse birth outcomes, which might further indicate a selection towards a low-risk population. To assess whether a screening model improved by adding additional maternal, fetal or placental characteristics, we assessed if changes in AUC effect estimates of different screening models were clinically relevant and whether the differences in AUCs of two different models were statistically significant. What is considered clinically relevant may be arbitrary. Based on previous studies focused on screening for similar adverse birth outcomes, we considered an approximate 4–5% change in effect estimate of the AUC as clinically relevant, as this change is likely associated with a detectable increase in sensitivity [14, 28, 44]. Next, when model comparison fulfilled this criterion, we used a statistical test by DeLong et al. to see if this change was statistically significant [32]. This method takes into account two correlated AUCs, which is necessary as two curves are constructed based on the same individuals. We included common maternal characteristics, easily available within all pregnant women and applicable to low-risk pregnant populations, within our maternal screening model. Another predictor for preterm birth, SGA or LGA at birth is occurrence of either of these outcomes in a previous pregnancy. We did not use this maternal characteristic for screening in our models, as women with a previous preterm birth, SGA or LGA newborn are already considered higher risk pregnant women and often intensified monitoring and additional ultrasounds for fetal growth are indicated. Among higher-risk populations, a different third trimester ultrasound screening model including other maternal characteristics may be more applicable or even a separate screening model for nulliparous and multiparous women may be needed. Further studies assessing screening performance for adverse birth outcomes of third trimester fetal and placental ultrasound, in combination with more maternal characteristics such as previous pregnancy complications, among high-risker populations are needed. All ultrasound measurements were performed according to the study protocol and blinded with regard to pregnancy outcomes due to the prospective nature of the study. Abnormal research ultrasound results were reported to healthcare providers and some participants might have been treated as a consequence of abnormal (research) ultrasound findings, which might have affected the screening performance. For example, if an abnormal EFW in a research ultrasound was found, this may have led to induction of labour before 37 weeks of gestation, which is considered iatrogenic preterm birth. However, when we restricted our analyses to spontaneous preterm birth only, we found similar screening performance. Thus, the performance of our model screening for preterm birth does not seem to be driven by iatrogenic preterm birth.

## Conclusion

Maternal characteristics together with single third trimester fetal and placental ultrasound has the best screening performance for preterm birth, and SGA and LGA at birth, compared to using only second trimester ultrasound or combined second and third trimester ultrasound. Compared to second trimester ultrasound screening, third trimester ultrasound screening would nearly double detection of fetuses at risk of these common adverse birth outcomes in low-risk populations.

## Supplementary information

Additional file 1 Index supplemental material.

Additional file 2 STROBE checklist.
