# Supplementary material for: Second and third trimester fetal ultrasound population screening for risks of preterm birth and small-size and large-size for gestational age at birth: a population-based prospective cohort study: Fetal ultrasound screening for common adverse birth outcomes
Source: BMC Med. 2020 Apr 7;18:63. doi: 10.1186/s12916-020-01540-x (PMC7137302; doi:10.1186/s12916-020-01540-x)
Supplement: Supplementary file 1 — Index supplemental material. [file 12916_2020_1540_MOESM1_ESM.docx]

**Index supplemental material**

| **Page** |  | |
| --- | --- | --- |
| **2** | Table S1 | Fetal and placental characteristics (N = 7670) |
| **3** | Table S2 | Characteristics of mothers and their children with and without placenta measures available |
| **4** | Table S3 | Statistical significance level of comparison of screening models with and without placenta measures for screening for preterm birth, small-size and large-size for gestational age |
| **4** | Table S4 | Estimated sensitivity at different levels of specificity for models screening for preterm birth, small-size and large-size for gestational age |
| **5** | Figure S1 | Flowchart population for analysis |
| **6** | Figure S2 | Receiver operating characteristic curves for models screening for spontaneous preterm birth |
| **7** | Figure S3 | Receiver operating characteristic curves for models screening for preterm birth, small-size and large-size for gestational age, with screen positive defined as estimated fetal weight <5th percentile, or >95th percentile |
| **8** | Figure S4 | Receiver operating characteristic curves for models screening for preterm birth, small-size and large-size for gestational age using abdominal circumference instead of estimated fetal weight |
| **9** | Figure S5 | Receiver operating characteristic curves for screening for preterm birth, small-size and large-size for gestational age, with separate umbilical and uterine artery indices |
| **10** | Figure S6 | Receiver operating characteristic curves for models screening for preterm birth, small-size and large-size for gestational age based on estimated fetal weight, without maternal characteristics |
| **11** | Figure S7 | Defining screen positive separately for small- and large-size for gestational age |
| **13** | Figure S8 | Receiver operating characteristic curves for models screening for small-size and large-size for gestational age according to size at birth <5th percentile, or >95th percentile |
| **15** | Figure S9 | Receiver operating characteristic curves for models screening for small-size and large-size for gestational age at birth defined as birth weight <3rd and >97th percentile, respectively |
| **17** | Figure S10 | Receiver operating characteristic curves for models screening for small-size and large-size for gestational age at birth with adverse outcomes |

**Table S1. Fetal and placental characteristics (N = 7670)**

| **Second trimester fetal and placental characteristics** |  |
| --- | --- |
| Gestational age in weeks, median (IQR) | 20.5(19.9 to 21.3) |
| Head circumference, mean (SD) (mm) | 179.5(14.7) |
| Abdominal circumference, mean (SD) (mm) | 157(15) |
| Femur length, mean (SD) (mm) | 33.5(3.6) |
| Estimated fetal weight, mean (SD) (g) | 382.3(95.5) |
| Estimated fetal weight < 10^th^ percentile (<-1.32SDS) No. (%) | 767(10) |
| Estimated fetal weight > 90^th^ percentile (>1.19SDS) No. (%) | 767(10) |
| Uterine artery resistance index, mean (SD) | 0.54(0.09) |
| Umbilical artery pulsatility index, mean (SD) | 1.2(0.19) |
| **Third trimester fetal and placental characteristics** |  |
| Gestational age in weeks, median (IQR) | 30.4(29.8 to 30.9) |
| Head circumference, mean (SD) (mm) | 284.9(12.4) |
| Abdominal circumference, mean (SD) (mm) | 263.8(16.5) |
| Femur length, mean (SD) (mm) | 57.5(3) |
| Estimated fetal weight, mean (SD) (g) | 1615.9(256.2) |
| Estimated fetal weight < 10^th^ percentile (<-1.20SDS), No. (%) | 767(10) |
| Estimated fetal weight > 90^th^ percentile (>1.35SDS) No. (%) | 767(10) |
| Uterine artery resistance index | 0.48(0.08) |
| Umbilical artery pulsatility index | 0.98(0.17) |
| Second to third trimester estimated fetal weight change <10^th^ percentile (<1.10SDS) No (%) | 767(10) |
| Second to third trimester estimated fetal weight change >90^th^ percentile (>1.35SDS) No (%) | 767(10) |

Abbreviations: IQR: inter quartile range; SD: Standard deviation;
Values are observed data and represent means (SD), medians (IQR) or number of subjects (valid %).

**Table S2. Characteristics of mothers and their children with and without placenta measures available^a^**

|  | **Placenta measures available N=7014^b^** | **No placenta measures available N=656^c^** | **P-value^d^** |
| --- | --- | --- | --- |
| **Maternal characteristics** |  |  |  |
| Age |  |  | 0.13 |
| < 25, No (%) | 1419(20.5) | 154(23.5) |  |
| 25-35, No (%) | 4559(65) | 413(63) |  |
| > 35, No (%) | 1036(14.8) | 89(13.6) |  |
| Height, mean (SD) (cm) | 167.3(7.36) | 166.6(7.4) | 0.013 |
| Weight, mean (SD) (kg) | 69(13) | 721(14.4) | <0.01 |
| Body Mass Index, mean (SD) (kg/m^2^) |  |  | <0.01 |
| Normal, No (%) | 4359(62.5) | 350(54.2) |  |
| Overweight, No (%) | 1809(25.9) | 170(26.3) |  |
| Obese, No (%) | 806(11.6) | 126(19.5) |  |
| Education, No. higher education (%) | 2837(43.6) | 218(36.2) | <0.01 |
| Race / Ethnicity, No. (%) |  |  | 0.11 |
| Dutch or European, No (%) | 3949(58.5) | 340(55) |  |
| Surinamese , No (%) | 587(8.7) | 68(11) |  |
| Turkish, No (%) | 622(9.2) | 51(8.3) |  |
| Moroccan , No (%) | 428(6.3) | 45(7.3) |  |
| Cape Verdian or Dutch Antilles, No (%) | 506(7.5) | 54(8.8) |  |
| Parity, No. nulliparous (%) | 3951(56.8) | 357(54.7) | 0.06 |
| Smoking, No. (%) |  |  | 0.19 |
| None, No (%) | 4532(72.6) | 435(75) |  |
| Early-pregnancy only, No (%) | 556(8.9) | 39(6.7) |  |
| Continued, No (%) | 1155(18.5) | 106(18.3) |  |
| **Birth characteristics** |  |  |  |
| Males, No. (%) | 3549(50.6) | 312(47.6) | 0.14 |
| Gestational age, median (IQR), weeks | 40.1(39.1 to 41.0) | 40(39.0 to 41.0) | 0.07 |
| Birth weight, mean (SD) grams | 3424(542) | 3419(567) | 0.83 |
| Preterm birth^e^, No. (%) | 307(4.4) | 38(5.8) | 0.09 |
| Small for gestational age^e^, No. (%) | 693(9.9) | 75(11.4) | 0.08 |
| Large for gestational age^e^, No. (%) | 689(9.8) | 78(11.9) | 0.08 |
| Caesarean delivery, No. (%) | 740(11.6) | 82(13.6) | 0.67 |
| Assisted vaginal delivery, No. (%) | 889(13.9) | 75(12.5) | 0.67 |
| Apgar score below 7 at 5 minutes, No. (%) | 73(0.8) | 5(0.8) | 0.75 |

Abbreviations: IQR: inter quartile range; SD: Standard deviation.

^a^ Because these measurements were only performed in one of two research centers, second and third trimester uterine artery resistance indices were available in subgroups of n=4,361 and n=4,193 women, respectively. Second and third trimester UA-PI were available in n=5,831 and n=6,224 women, respectively.
^b^ Participants with any of the following placenta measures available: 2^nd^ or 3^rd^ trimester umbilical artery pulsatility index or 2^nd^ or 3^rd^ trimester uterine artery resistance index. Values are observed data and represent means (SD), medians (IQR) or number of subjects (valid %).
^c^ Participants without any of the following placenta measures available: 2^nd^ or 3^rd^ trimester umbilical artery pulsatility index or 2^nd^ or 3^rd^ trimester uterine artery resistance index. Values are observed data and represent means (SD), medians (IQR) or number of subjects (valid %).
^d^ P-value for difference between groups.
^e^ Preterm birth is defined as birth before 37 weeks; Small-size for gestational age is defined as < 10^th^ percentile (<-1.4 SDS) of gestational age-and sex-adjusted birth weight; Large-size for gestational age is defined as > 90^th^ percentile (>1.18 SDS) of gestational age-and sex-adjusted birth weight.

**Table S3. Statistical significance level of comparison of screening models with and without placenta measures, for screening for preterm birth, small-size and large-size for gestational age**

|  | **Preterm birth** | **Small-size for gestational age** | **Large-size for gestational age** |
| --- | --- | --- | --- |
| Maternal characteristics vs. third trimester model | P<0.01 | P<0.01 | P<0.01 |
| Second trimester model vs. third trimester model | P<0.01 | P<0.01 | P<0.01 |
|  |  |  |  |
| Third trimester model vs. Third trimester model and placenta measures* | P<0.01 | P<0.01 | NA |

Models are based on maternal characteristics, estimated fetal weight, and placenta measures. NA: Not applicable as no comparison was made. Values are p-values for comparison of models, using the method of DeLong et.al for comparison of two correlated AUCs. *Analyses performed in a subsample with all placenta measures available.

**Table S4. Estimated sensitivity at different levels of specificity for models screening for preterm birth, small-size and large-size for gestational age**

|  |  | **Estimated sensitivities** | |
| --- | --- | --- | --- |
|  | **Specificity** | **Maternal characteristics model** | **Second trimester model^a^** |
| **Preterm birth** | 70% | 44% | 44% |
|  | 80% | 33% | 32% |
|  | 90% | 18% | 19% |
| **Small-size for gestational age** | 70% | 56% | 65% |
|  | 80% | 43% | 51% |
|  | 90% | 25% | 33% |
| **Large-size for gestational age** | 70% | 53% | 58% |
|  | 80% | 43% | 44% |
|  | 90% | 25% | 28% |

Values are estimated sensitivity at different levels of specificity. Derived from receiver operating characteristic curve. ^a^Second trimester model: Maternal characteristics and 2^nd^ trimester estimated fetal weight.

**Figure S1. Flowchart population for analysis**

**n** **=** **8879**Participants enrolled during pregnancy

n = 1130 excluded due to no second and third trimester fetal growth measurements

**n** **=** **7749**Participants eligible for present study

n = 33 excluded due to fetal deaths (n = 15), twin births (n = 2) and loss to follow-up (n = 16)

**n** **=** **7716**Participants with singleton live births eligible for present study

n = 46 excluded due to no information of birth outcomes available (weight and / or gestational age at birth)

**Total population for analysis n = 7670**

**Second trimester**Head circumference n = 7613
Abdominal Circumference n = 7670
Femur length n = 7670
Estimated fetal weight n = 7670
Uterine artery resistance index n = 4361
Umbilical artery pulsatility index n = 5831

**Third trimester**Head circumference n = 7596
Abdominal Circumference n = 7670
Femur length n = 7670
Estimated fetal weight n = 7670
Uterine artery resistance index n = 4193
Umbilical artery pulsatility index n = 6224

**Birth**Gestational age at birth n = 7670
Weight n = 7670

**Figure S2. Receiver operating characteristic curves for models screening for spontaneous preterm birth^a^**

| **Screening performance for spontaneous preterm birth based**  **on maternal and fetal characteristics^b^** | | | **Screening performance for spontaneous preterm birth based**  **on maternal, fetal and placental characteristics^c^** | | |
| --- | --- | --- | --- | --- | --- |
| 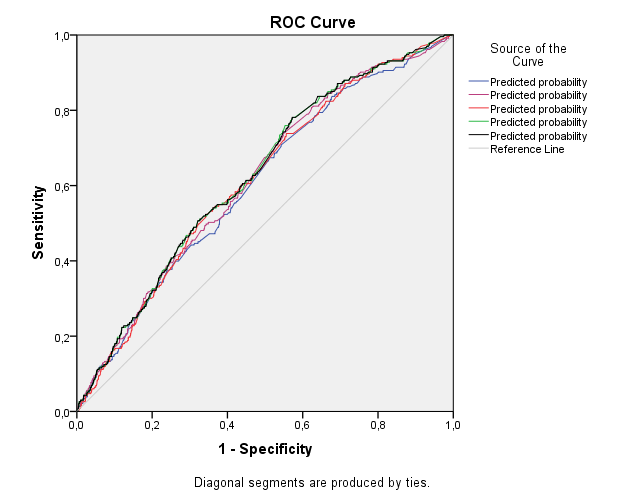 | **Models** | **AUC**  **(95% Confidence Interval)** | 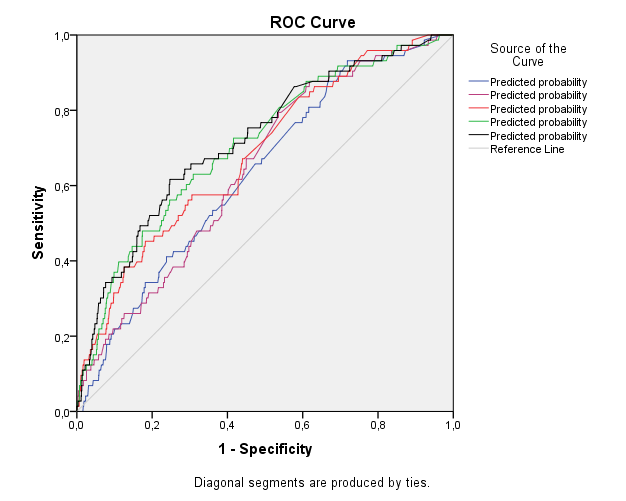 | **Models** | **AUC**  **(95% Confidence Interval)** |
|  | Maternal characteristics:  (Blue line) | 0.60 (0.57 to 0.64) |  | Maternal characteristics:  (Blue line) | 0.63 (0.57 to 0.69) |
|  | Second trimester:  (Purple line) | 0.62 (0.58 to 0.65) |  | Second trimester:  (Purple line) | 0.64 (0.59 to 0.70) |
|  | Third trimester:  (Red line) | 0.61 (0.58 to 0.65) |  | Third trimester:  (Red line) | 0.68 (0.62 to 0.74) |
|  | Second and third trimester:  (Green line) | 0.63 (0.59 to 0.66) |  | Second and third trimester:  (Green line) | 0.71 (0.65 to 0.77) |
|  | Growth:  (Black line) | 0.63 (0.59 to 0.66) |  | Growth:  (Black line) | 0.72 (0.66 to 0.78) |
|  |  | |  |  | |

^a^ Receiver operating characteristic curves and corresponding area under the curve (95% confidence interval) of models based on predicted values from the five models for screening for spontaneous preterm birth. Spontaneous preterm birth was defined as birth before 37^th^ week of gestation, after non-medically induced labor.
^b^ Model characteristics: Maternal characteristics; Second trimester model: Maternal characteristics and 2^nd^ trimester EFW; Third trimester model: Maternal characteristics and 3^rd^ trimester EFW; Second and third trimester model: Maternal characteristics, 2^nd^ and 3^rd^ trimester EFW; Growth model: Maternal characteristics, 2^nd^ and 3^rd^ trimester EFW and 2^nd^ to 3^rd^ trimester EFW change.
^c^ AUCs based on a sample of 2705 participants with all placenta measures available. EFW models as described under ^b^ with additional placenta measures. Second trimester model: 2^nd^ trimester uterine artery resistance index and 2^nd^ trimester umbilical artery pulsatility index. Third trimester model; 3^rd^ trimester uterine artery resistance index and 3^rd^ trimester umbilical artery pulsatility index; Second and third trimester model: 2^nd^ and 3^rd^  trimester uterine artery resistance index, and 2^nd^ and 3^rd^ trimester umbilical artery pulsatility index; Growth model: 2^nd^ and 3^rd^  trimester uterine artery resistance index and 2^nd^ and 3^rd^ trimester umbilical artery pulsatility index, 2^nd^ to 3^rd^ trimester change of uterine artery resistance index and umbilical artery pulsatility index.

**Figure S3. Receiver operating characteristic curves for models screening for preterm birth, small-size and large-size for gestational age, with screen positive defined as estimated fetal weight <5^th^ percentile, or >95^th^ percentile^a^**

| **Preterm birth** | | | | | |
| --- | --- | --- | --- | --- | --- |
| **Estimated fetal weight^b^** | | | | **Estimated fetal weight** **and placenta measures^c^** | |
| 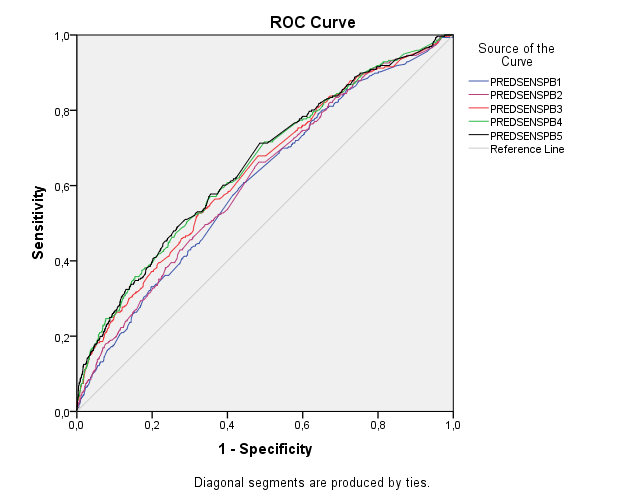 | | Model 1: 0.61 (0.58 to 0.64)  Model 2: 0.62 (0.58 to 0.65)  Model 3: 0.64 (0.61 to 0.67)  Model 4: 0.65 (0.62 to 0.69)  Model 5: 0.66 (0.62 to 0.69) | | 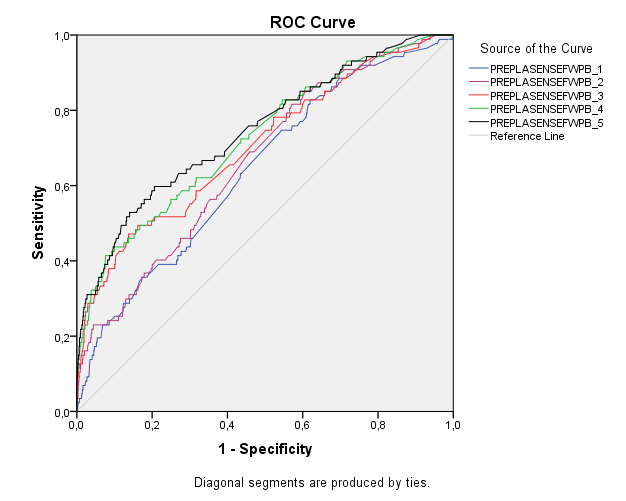 | Model 1: 0.64 (0.58 to 0.70)  Model 2: 0.67 (0.61 to 0.72)  Model 3: 0.71 (0.64 to 0.77)  Model 4: 0.73 (0.67 to 0.78)  Model 5: 0.75 (0.69 to 0.80) |
| **Small-size for gestational age** | | | | | |
| **Estimated fetal weight^b^** | | | | **Estimated fetal weight and placenta measures^c^** | |
| 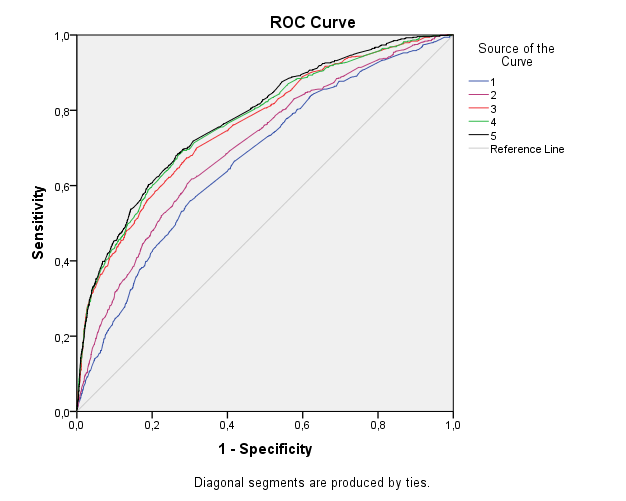 | | | Model 1: 0.67 (0.65 to 0.69)  Model 2: 0.70 (0.68 to 0.72)  Model 3: 0.76 (0.74 to 0.78)  Model 4: 0.77 (0.75 to 0.79)  Model 5: 0.78 (0.76 to 0.80) | 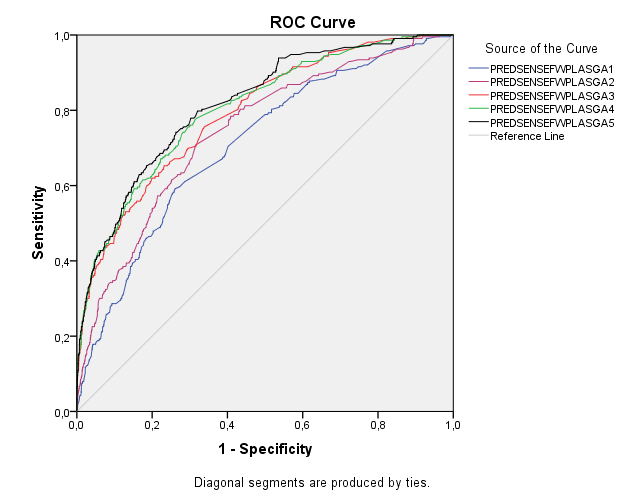 | Model 1: 0.71 (0.67 to 0.74)  Model 2: 0.74 (0.71 to 0.78)  Model 3: 0.79 (0.76 to 0.82)  Model 4: 0.81 (0.77 to 0.84)  Model 5: 0.82 (0.79 to 0.85) |
| **Large-size for gestational age** | | | | | |
| **Estimated fetal weight^b^** | | | | **Estimated fetal weight and placenta measures^c^** | |
| 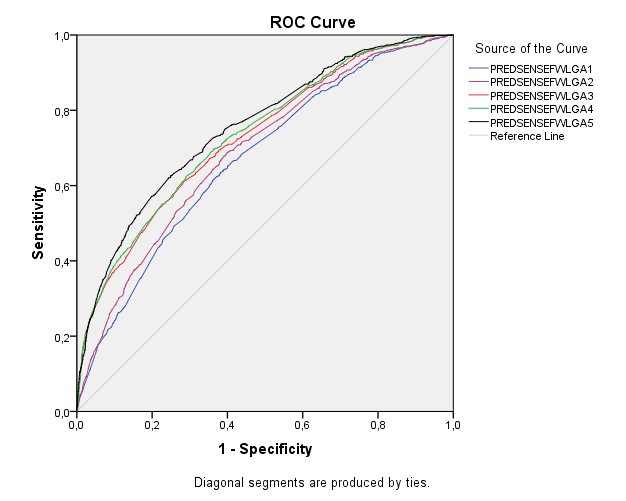 | Model 1: 0.67 (0.65 to 0.69)  Model 2: 0.69 (0.67 to 0.71)  Model 3: 0.73 (0.71 to 0.75)  Model 4: 0.73 (0.71 to 0.75)  Model 5: 0.75 (0.73 to 0.77) | | | **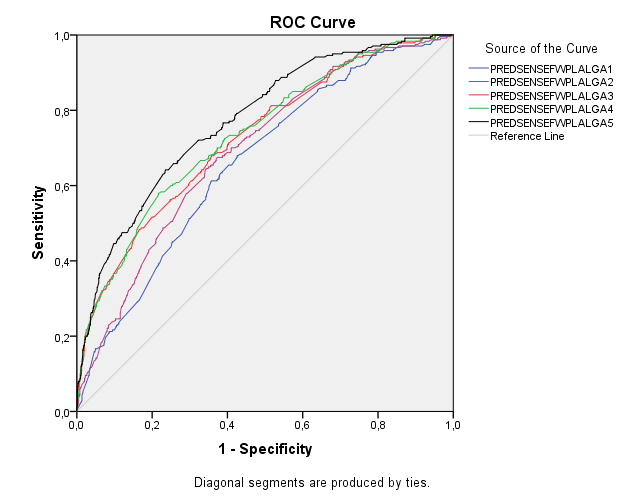** | Model 1: 0.66 (0.63 to 0.70)  Model 2: 0.70 (0.66 to 0.73)  Model 3: 0.73 (0.69 to 0.76)  Model 4: 0.74 (0.70 to 0.77)  Model 5: 0.77 (0.74 to 0.81) |

^a^ Receiver operating characteristic curves and corresponding area under the curve (95% CI) based on predicted values from five models for screening for preterm birth, small- and large-size for gestational age. Models are based on maternal characteristics, second and third trimester fetal and placental ultrasound.
^b^ Model 1: Maternal characteristics; Model 2: Maternal characteristics and 2^nd^ trimester EFW; Model 3: Maternal characteristics and 3^rd^ trimester EFW; Model 4: Maternal characteristics, 2^nd^ +3^rd^ trimester EFW; Model 5: Maternal characteristics, 2^nd^ + 3^rd^ trimester EFW and 2^nd^ to 3^rd^ trimester EFW change.
^c^ AUCs based on a sample of 2705 participants with all placenta measures available. Models as EFW models under ^b^, adding the following placenta measures. Model 2: 2^nd^ trimester uterine artery resistance index, and 2^nd^ trimester umbilical artery pulsatility index. Model 3: 3^rd^ trimester uterine artery resistance index, and 3^rd^ trimester umbilical artery pulsatility index; Model 4: 2^nd^ and 3^rd^  trimester uterine artery resistance index, and 2^nd^ and 3^rd^ trimester umbilical artery pulsatility index; Model 5: 2^nd^ and 3^rd^  trimester uterine artery resistance index, and 2^nd^ and 3^rd^ trimester umbilical artery pulsatility index, 2^nd^ to 3^rd^ trimester change of uterine artery resistance index and umbilical artery pulsatility index.

Grey line: Reference line;
Blue line: Model 1: Maternal characteristics model;
Purple line: Model 2: Second trimester model;
Red line: Model 3: Third trimester model;
Green line: Model 4: Second and third trimester model;
Black line: Model 5: Growth model.

**Figure S4. Receiver operating characteristic curves for models screening for preterm birth, small-size and large-size for gestational age using abdominal circumference instead of estimated fetal weight^a^**

| **Preterm birth** | | | | | |
| --- | --- | --- | --- | --- | --- |
| **Abdominal circumference^b^** | | | **Abdominal circumference and placenta measures^c^** | | |
| 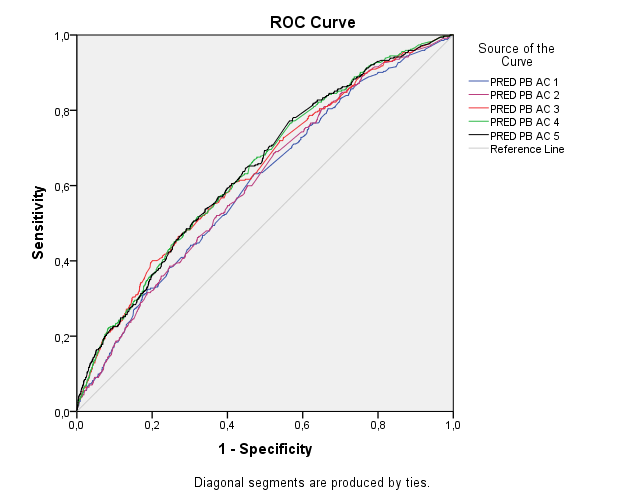 | Model 1: 0.60 (0.57 to 0.63)  Model 2: 0.61 (0.58 to 0.64)  Model 3: 0.64 (0.61 to 0.67)  Model 4: 0.64 (0.61 to 0.67)  Model 5: 0.64 (0.61 to 0.67) | | 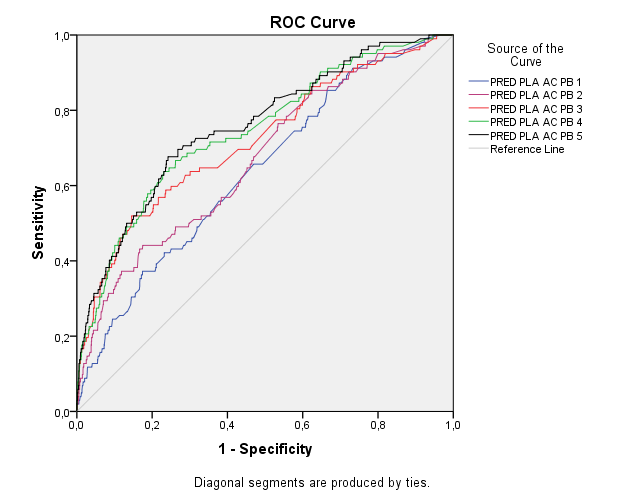 | Model 1: 0.64 (0.59 to 0.69)  Model 2: 0.67 (0.62 to 0.73)  Model 3: 0.72 (0.66 to 0.78)  Model 4: 0.75 (0.69 to 0.80)  Model 5: 0.76 (0.71 to 0.81) | |
| **Small-size for gestational age** | | | | | |
| **Abdominal circumference^b^** | | | **Abdominal circumference and placenta measures^c^** | | |
| 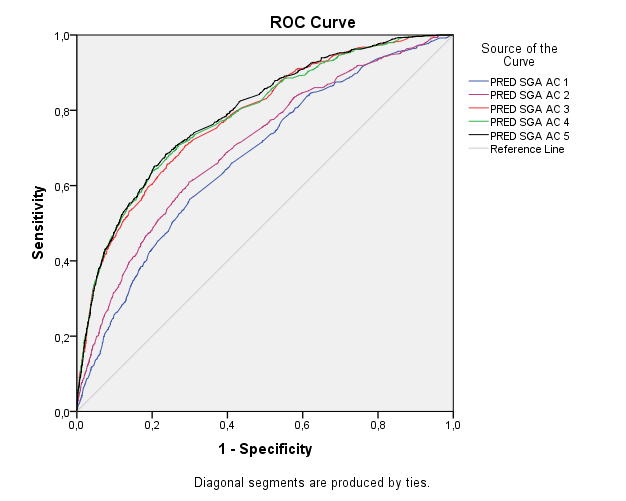 | Model 1: 0 67 (0.65 to 0.69)  Model 2: 0.70 (0.68 to 0.72)  Model 3: 0.78 (0.76 to 0.80)  Model 4: 0.79 (0.77 to 0.80)  Model 5: 0.79 (0.77 to 0.81) | | 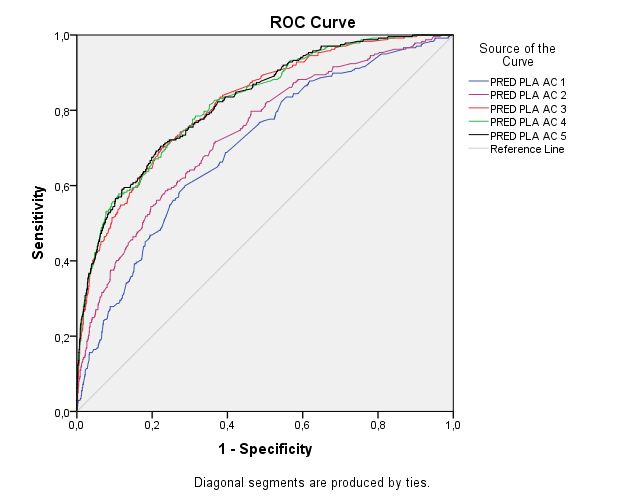 | Model 1: 0.70 (0.67 to 0.74)  Model 2: 0.74 (0.71 to 0.77)  Model 3: 0.82 (0.79 to 0.85)  Model 4: 0.82 (0.79 to 0.85)  Model 5: 0.82 (0.80 to 0.85) | |
| **Large-size for gestational age** | | | | | |
| **Abdominal circumference^b^** | | | **Abdominal circumference and placenta measures^c^** | | |
| 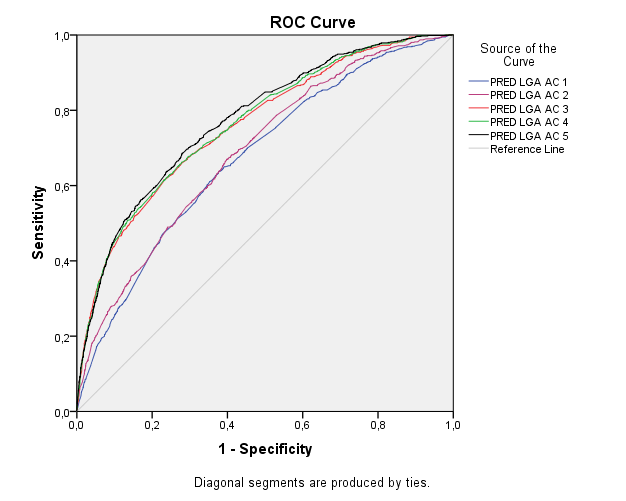 | | Model 1: 0.68 (0.66 to 0.70)  Model 2: 0.69 (0.67 to 0.71)  Model 3: 0.76 (0.74 to 0.78)  Model 4: 0.77 (0.75 to 0.78)  Model 5: 0.78 (0.76 to 0.79) | 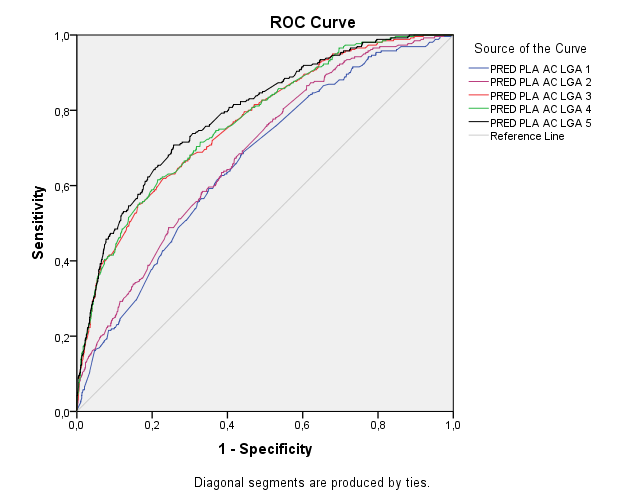 | | Model 1: 0.67 (0.63 to 0.70)  Model 2: 0.69 (0.65 to 0.72)  Model 3: 0.77 (0.74 to 0.80)  Model 4: 0.77 (0.74 to 0.80)  Model 5: 0.79 (0.76 to 0.82) |

^a^ Receiver operating characteristic curves of models based on predicted values from five models for preterm birth, small- and large-size for gestational age. Models based on fetal abdominal circumference, and placenta measures.
^b^ Model 1: Maternal characteristics; Model 2: Maternal characteristics and 2^nd^ trimester AC; Model 3: Maternal characteristics and 3^rd^ trimester AC; Model 4: Maternal characteristics, 2^nd^ +3^rd^ trimester AC; Model 5: Maternal characteristics, 2^nd^ + 3^rd^ trimester AC and 2^nd^ to 3^rd^ trimester AC change.
^c^ AUCs based on a sample of 2705 participants with all placenta measures available. Models as AC models under ^b^, adding the following placenta measures: Model 2: 2^nd^ trimester uterine artery resistance index and 2^nd^ trimester umbilical artery pulsatility index. Model 3: 3^rd^ trimester uterine artery resistance index and 3^rd^ trimester umbilical artery pulsatility index; Model 4: 2^nd^ and 3^rd^  trimester uterine artery resistance index, and 2^nd^ and 3^rd^ trimester umbilical artery pulsatility index; Model 5: 2^nd^ and 3^rd^  trimester uterine artery resistance index and 2^nd^ and 3^rd^ trimester umbilical artery pulsatility index, 2^nd^ to 3^rd^ trimester change of uterine artery resistance index and umbilical artery pulsatility index.

Grey line: Reference line;
Blue line: Model 1: Maternal characteristics model;
Purple line: Model 2: Second trimester model;
Red line: Model 3: Third trimester model;
Green line: Model 4: Second and third trimester model;
Black line: Model 5: Growth model

**Figure S5. Receiver operating characteristic curves for screening for preterm birth, small-size and large-size for gestational age, with separate umbilical and uterine artery indices^a^**

| **Estimated fetal weight and only umbilical artery pulsatility index^bc^** | | |
| --- | --- | --- |
| **Preterm birth**  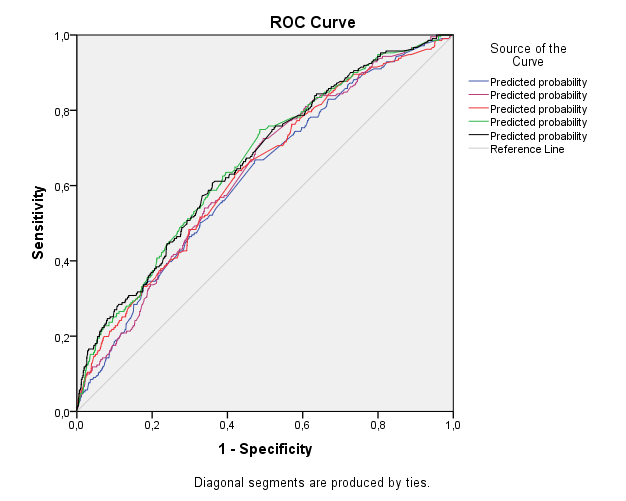  Model 1: 0.62 (0.59 to 0.66)  Model 2: 0.64 (0.60 to 0.67)  Model 3: 0.64 (0.60 to 0.67)  Model 4: 0.66 (0.63 to 0.70)  Model 5: 0.66 (0.63 to 0.70) | **Small-size for gestational age**  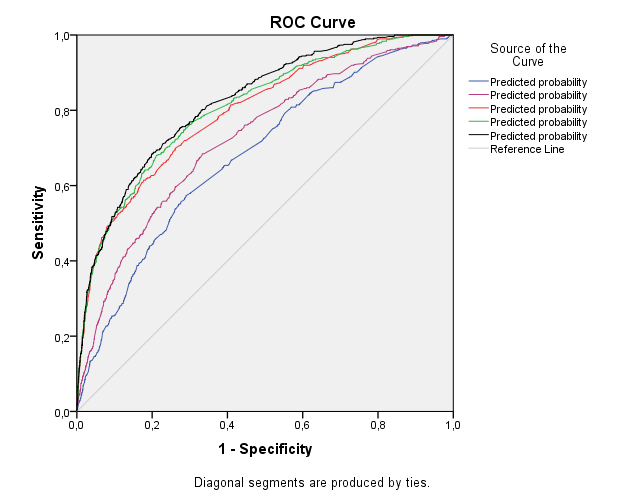  Model 1: 0.68 (0.66 to 0.71)  Model 2: 0.73 (0.70 to 0.75)  Model 3: 0.80 (0.78 to 0.75)  Model 4: 0.81 (0.79 to 0.83)  Model 5: 0.82 (0.80 to 0.84) | **Large-size for gestational age**  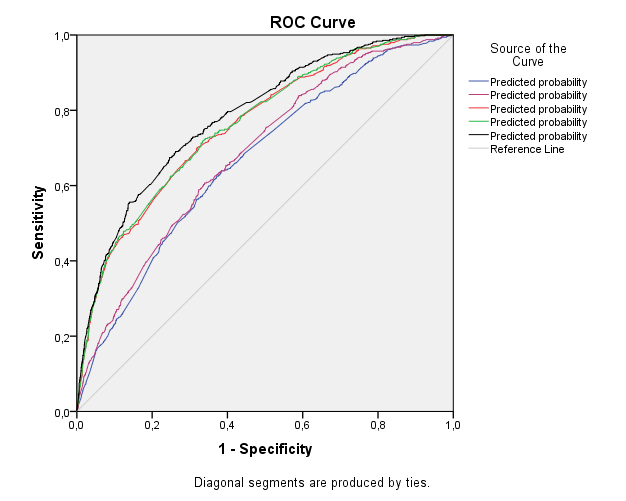  Model 1: 0.67 (0.64 to 0.69)  Model 2: 0.69 (0.66 to 0.71)  Model 3: 0.76 (0.74 to 0.78)  Model 4: 0.76 (0.74 to 0.79)  Model 5: 0.79 (0.76 to 0.81) |
|  | | |
| **Estimated fetal weight and only uterine artery resistance index^bd^** | | |
| **Preterm birth**  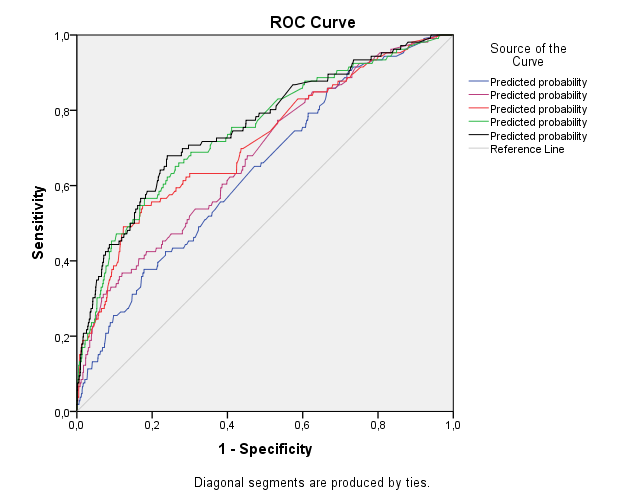  Model 1: 0.64 (0.59 to 0.69)  Model 2: 0.68 (0.63 to 0.73)  Model 3: 0.71 (0.66 to 0.77)  Model 4: 0.75 (0.69 to 0.80)  Model 5: 0.76 (0.71 to 0.81) | **Small-size for gestational age**  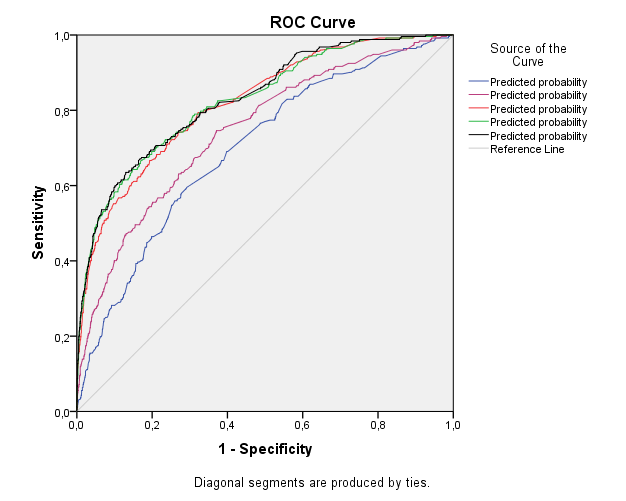  Model 1: 0.70 (0.66 to 0.73)  Model 2: 0.75 (0.71 to 0.78)  Model 3: 0.82 (0.79 to 0.85)  Model 4: 0.83 (0.80 to 0.85)  Model 5: 0.83 (0.80 to 0.86) | **Large-size for gestational age**  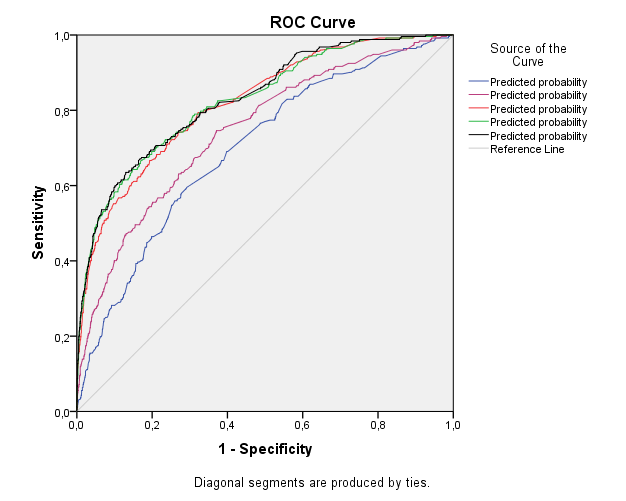  Model 1: 0.70 (0.66 to 0.73)  Model 2: 0.75 (0.71 to 0.78)  Model 3: 0.82 (0.79 to 0.85)  Model 4: 0.83 (0.80 to 0.85)  Model 5: 0.83 (0.80 to 0.86) |

^a^ Receiver operating characteristic curves of models based on EFW combined with umbilical artery pulsatility index or uterine artery pulsatility index. Values are area under the curve (95% confidence interval).
^b^ Model 1: Maternal characteristics; Model 2: Maternal characteristics and 2^nd^ trimester EFW; Model 3: Maternal characteristics and 3^rd^ trimester EFW; Model 4: Maternal characteristics, 2^nd^ +3^rd^ trimester EFW; Model 5: Maternal characteristics, 2^nd^ + 3^rd^ trimester EFW and 2^nd^ to 3^rd^ trimester EFW change.
^c^ EFW models as described under ^b^ with additional placenta measures. Second trimester model: 2^nd^ trimester umbilical artery pulsatility index. Third trimester model; 3^rd^ trimester umbilical artery pulsatility index; Second and third trimester model: 2^nd^ and 3^rd^ trimester umbilical artery pulsatility index; 2^nd^ and 3^rd^ trimester umbilical artery pulsatility index, 2^nd^ to 3^rd^ trimester change of umbilical artery pulsatility index.
^d^ EFW models as described under ^b^ with additional placenta measures. Second trimester model: 2^nd^ trimester uterine artery resistance index; Third trimester model; 3^rd^ trimester uterine artery resistance index; Second and third trimester model: 2^nd^ and 3^rd^  trimester uterine artery resistance index; Growth model: 2^nd^ and 3^rd^  trimester uterine artery resistance index and 2^nd^ to 3^rd^ trimester change of uterine artery resistance index.

Grey line: Reference line;
Blue line: Model 1: Maternal characteristics model;
Purple line: Model 2: Second trimester model;
Red line: Model 3: Third trimester model;
Green line: Model 4: Second and third trimester model;
Black line: Model 5: Growth model.

**Figure S6. Receiver operating characteristic curves for models screening for preterm birth, small-size and large-size for gestational age based on estimated fetal weight, without maternal characteristics^ab^**

| **Preterm birth** | **Small-size for gestational age** | **Large-size for gestational age** |
| --- | --- | --- |
| 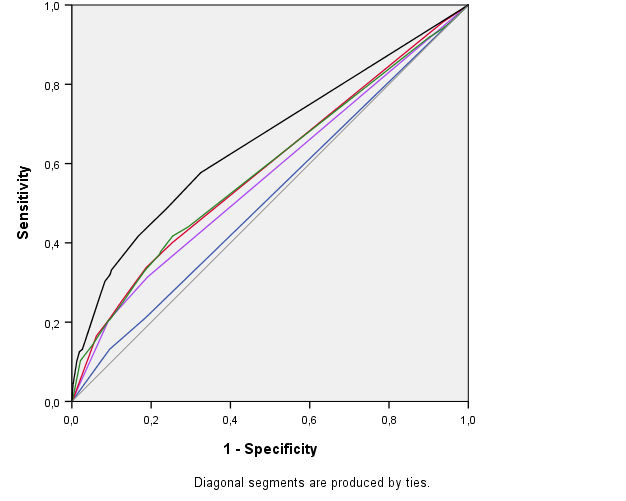  Model 2: 0.54 (0.50 to 0.57)  (blue)  Model 3: 0.57 (0.54 to 0.60)  (purple)  Model 3^c^: 0.66 (0.61 to 0.70)  (black)  Model 4: 0.59 (0.56 to 0.63)  (red)  Model 5: 0.60 (0.57 to 0.63)  (green) | 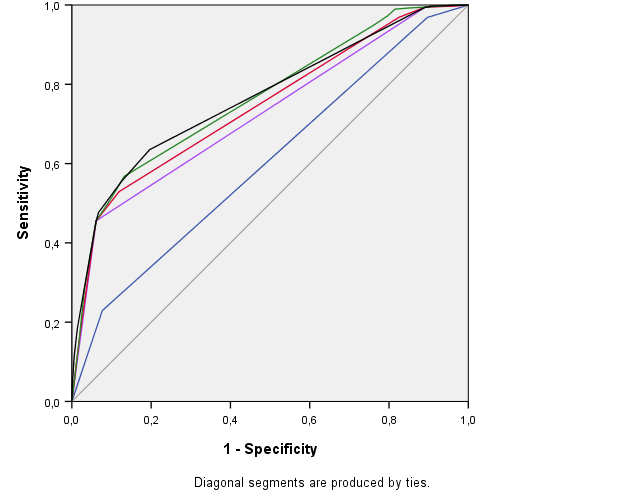Model 2: 0.61 (0.59 to 0.63)  (blue)  Model 3: 0.71 (0.69 to 0.73)  (purple)  Model 3 ^c^: 0.77 (0.74 to 0.80)  (black)  Model 4: 0.74 (0.72 to 0.76)  (red)  Model 5: 0.76 (0.74 to 0.78)  (green) | 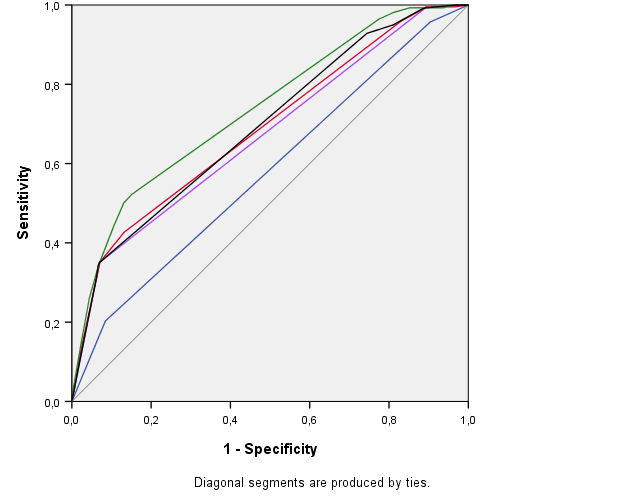Model 2: 0.58 (0.56 to 0.60)  (blue)  Model 3: 0.68 (0.65 to 0.70)  (purple)  Model 3 ^c^: 0.69 (0.67 to 0.72)  (black)  Model 4: 0.69 (0.67 to 0.71)  (red)  Model 5: 0.73 (0.71 to 0.75)  (green) |

^a^ Receiver operating characteristic curves and corresponding area under the curve (95% CI) based on predicted values from four models for preterm birth, small- and large-size for gestational age. Models are based on second and third trimester fetal ultrasound.
^b^ Model 2: 2^nd^ trimester EFW; Model 3: 3^rd^ trimester EFW; Model 4: 2^nd^ +3^rd^ trimester EFW; Model 5: 2^nd^ + 3^rd^ trimester EFW and 2^nd^ to 3^rd^ trimester EFW change.
^c^ AUCs based on a sample of 2705 participants with all placenta measures available. Models 3^c^ 3^rd^ trimester EFW + 3^rd^ trimester uterine artery resistance index and 3^rd^ trimester umbilical artery pulsatility index;

**Figure S7. Defining screen positive separately for small- and large-size for gestational age^a^.**

| **Screening performance for small-size for gestational age based**  **on maternal and fetal characteristics**^b,c^ | | | **Screening performance for small-size for gestational age based**  **on maternal, fetal and placental characteristics**^c,d^ | | |
| --- | --- | --- | --- | --- | --- |
| 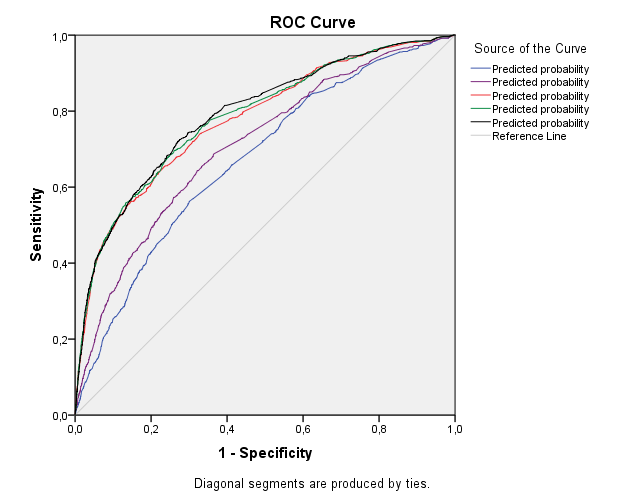 | **Models** | **AUC (95% Confidence Interval)** | 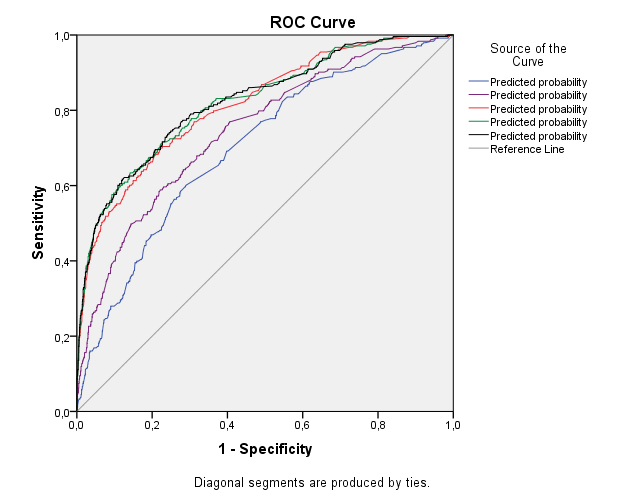 | **Models** | **AUC (95% Confidence Interval)** |
|  | Maternal characteristics:  (Blue line) | 0.67 (0.65 to 0.69) |  | Maternal characteristics:  (Blue line) | 0.70 (0.67 to 0.74) |
|  | Second trimester:  (Purple line) | 0.71 (0.69 to 0.73) |  | Second trimester:  (Purple line) | 0.75 (0.71 to 0.78) |
|  | Third trimester:  (Red line) | 0.78 (0.76 to 0.80) |  | Third trimester:  (Red line) | 0.82 (0.79 to 0.85) |
|  | Second and third trimester:  (Green line) | 0.89 (0.77 to 0.80) |  | Second and third trimester:  (Green line) | 0.82 (0.79 to 0.85) |
|  | Growth:  (Black line) | 0.79 (0.77 to 0.81) |  | Growth:  (Black line) | 0.82 (0.80 to 0.85) |
|  |  |  |  |  |  |
|  | | |  | | |
| **Sensitivity of the third-trimester-models based on estimated fetal weight** | | | **Sensitivity of the third trimester models based on EFW and placenta measures** | | |
| **Specificity** |  | | **Specificity** |  | |
| 70% | 71% | | 70% | 75% | |
| 80% | 61% | | 80% | 66% | |
| 90% | 49% | | 90% | 54% | |
|  |  | |  |  | |
| **Screening performance for large-size for gestational age based**  **on maternal and fetal characteristics**^b,e^ | | | **Screening performance for large-size for gestational age based**  **on maternal, fetal and placental characteristics**^d,e^ | | |
| 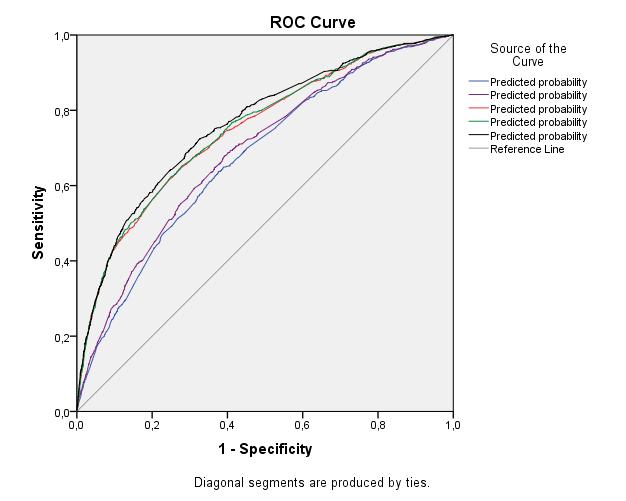 | **Models** | **AUC (95% Confidence Interval)** | 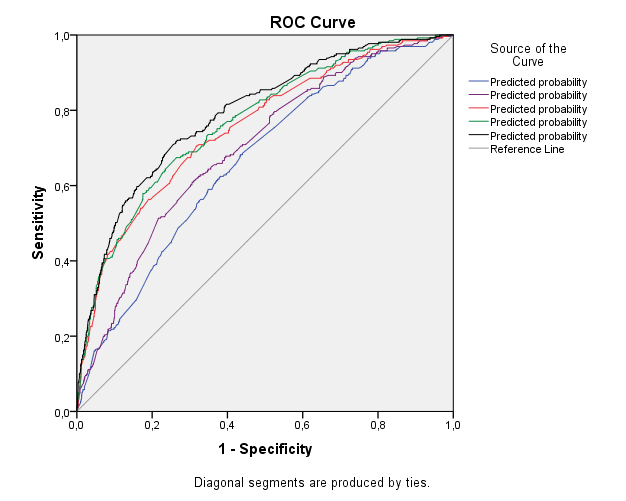 | **Models** | **AUC (95% Confidence Interval)** |
|  | Maternal characteristics:  (Blue line) | 0.68 (0.66 to 0.70) |  | Maternal characteristics:  (Blue line) | 0.67 (0.63 to 0.70) |
|  | Second trimester:  (Purple line) | 0.69 (0.67 to 0.71) |  | Second trimester:  (Purple line) | 0.70 (0.67 to 0.79) |
|  | Third trimester:  (Red line) | 0.75 (0.73 to 0.77) |  | Third trimester:  (Red line) | 0.76 (0.72 to 0.79) |
|  | Second and third trimester:  (Green line) | 0.75 (0.73 to 0.77) |  | Second and third trimester:  (Green line) | 0.77 (0.74 to 0.80) |
|  | Growth:  (Black line) | 0.76 (0.74 to 0.78) |  | Growth:  (Black line) | 0.79 (0.76 to 0.82) |
|  |  |  |  |  |  |
|  | | |  | | |
| **Sensitivity of the third-trimester-models based on estimated fetal weight** | | | **Sensitivity of the third trimester models based on EFW and placenta measures** | | |
| **Specificity** |  | | **Specificity** |  | |
| 70% | 67% | | 70% | 67% | |
| 80% | 57% | | 80% | 57% | |
| 90% | 43% | | 90% | 45% | |

AUC: area under the curve; CI: Confidence interval; EFW: Estimated fetal weight;

^a^ Receiver operating characteristic curve for the detection of fetuses at risk for small-size and large-size for gestational age based on second and third trimester fetal ultrasound and placenta measures, and derived sensitivity and specificity of the third trimester model.
^b^ Model characteristics: Maternal characteristics; Second trimester model: Maternal characteristics and 2^nd^ trimester EFW; Third trimester model: Maternal characteristics and 3^rd^ trimester EFW; Combined model: Maternal characteristics, 2^nd^ and 3^rd^ trimester EFW; Growth model: Maternal characteristics, 2^nd^ and 3^rd^ trimester EFW and 2^nd^ to 3^rd^ trimester EFW change.
^c^ Screen positive is defined as EFW <10^th^ percentile.
^d^ AUCs based on a sample of 2705 participants with all placenta measures available. Models are EFW models as described under ^b^ with additional placenta measures. Second trimester model: 2^nd^ trimester uterine artery resistance index and 2^nd^ trimester umbilical artery pulsatility index. Third trimester model; 3^rd^ trimester uterine artery resistance index and 3^rd^ trimester umbilical artery pulsatility index; Second and third trimester model: 2^nd^ and 3^rd^  trimester uterine artery resistance index, and 2^nd^ and 3^rd^ trimester umbilical artery pulsatility index; Growth model: 2^nd^ and 3^rd^  trimester uterine artery resistance index and 2^nd^ and 3^rd^ trimester umbilical artery pulsatility index, 2^nd^ to 3^rd^ trimester change of uterine artery resistance index and umbilical artery pulsatility index.
^e^ Screen positive is defined as EFW >90^th^ percentile.

**Figure S8. Receiver operating characteristic curves for models screening for small-size and large-size for gestational age according to size at birth <5^th^ percentile, or >95^th^ percentile^a^**

| **Screening performance for small-size for gestational age based**  **on maternal and fetal characteristics**^b^ | | | **Screening performance for small-size for gestational age based**  **on maternal, fetal and placental characteristics^c^** | | |
| --- | --- | --- | --- | --- | --- |
| 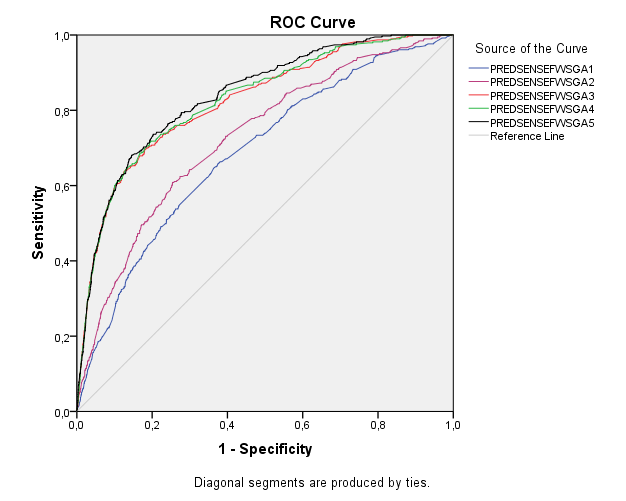 | **Models** | **AUC (95% Confidence Interval)** | 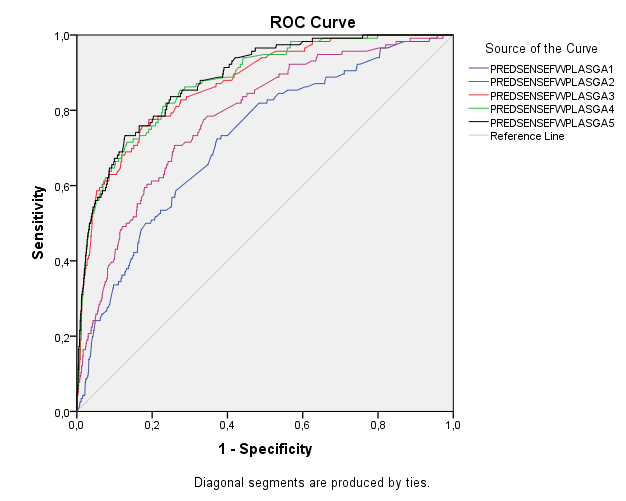 | **Models** | **AUC (95% Confidence Interval)** |
|  | Maternal characteristics:  (Blue line) | 0.69 (0.66 to 0.71) |  | Maternal characteristics:  (Blue line) | 0.72 (0.68 to 0.77) |
|  | Second trimester:  (Purple line) | 0.73 (0.70 to 0.75) |  | Second trimester:  (Purple line) | 0.78 (0.74 to 0.82) |
|  | Third trimester:  (Red line) | 0.82 (0.80 to 0.85) |  | Third trimester:  (Red line) | 0.87 (0.84 to 0.90) |
|  | Second and third trimester:  (Green line) | 0.83 (0.81 to 0.85) |  | Second and third trimester:  (Green line) | 0.88 (0.85 to 0.91) |
|  | Growth:  (Black line) | 0.84 (0.82 to 0.86) |  | Growth:  (Black line) | 0.88 (0.85 to 0.91) |
|  | | |  | | |
| **Sensitivity of the third trimester models based on EFW** ^d^ | | | **Sensitivity of the third trimester models based on EFW and placenta measures** ^d^ | | |
| **Specificity** | **SGA if gestational age adjusted birthweight <p5** | | **Specificity** | **SGA if gestational age adjusted birthweight <p5** | |
| 70% | 77% | | 70% | 84% | |
| 80% | 71% | | 80% | 78% | |
| 90% | 59% | | 90% | 63% | |
|  |  | |  |  | |
| **Screening performance for large-size for gestational age based**  **on maternal and fetal characteristics**^b^ | | | **Screening performance for large-size for gestational age based**  **on maternal, fetal and placental characteristics^c^** | | |
| 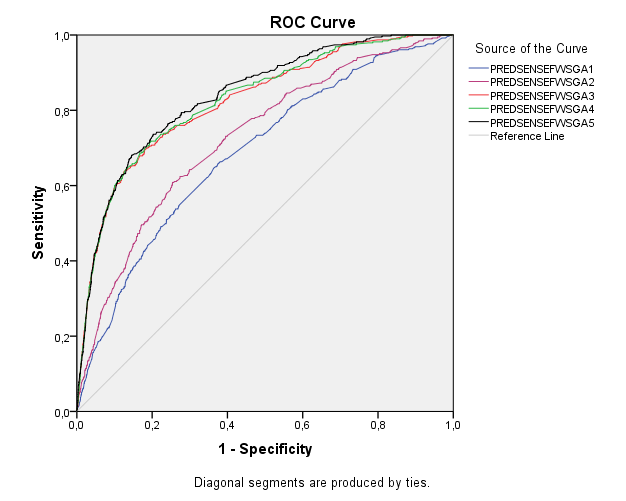 | **Models** | **AUC (95% Confidence Interval)** | 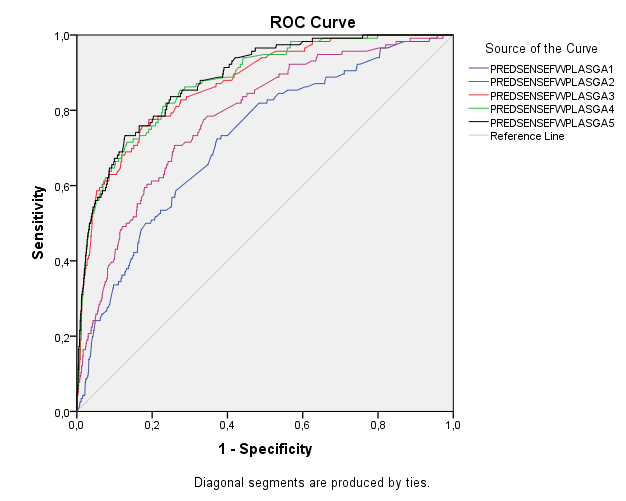 | **Models** | **AUC (95% Confidence Interval)** |
|  | Maternal characteristics:  (Blue line) | 0.69 (0.67 to 0.72) |  | Maternal characteristics:  (Blue line) | 0.67 (0.62 to 0.72) |
|  | Second trimester:  (Purple line) | 0.72 (0.69 to 0.74) |  | Second trimester:  (Purple line) | 0.72 (0.68 to 0.77) |
|  | Third trimester:  (Red line) | 0.79 (0.76 to 0.81) |  | Third trimester:  (Red line) | 0.78 (0.74 to 0.82) |
|  | Second and third trimester:  (Green line) | 0.79 (0.77 to 0.81) |  | Second and third trimester:  (Green line) | 0.80 (0.76 to 0.84) |
|  | Growth:  (Black line) | 0.81 (0.79 to 0.83) |  | Growth:  (Black line) | 0.82 (0.79 to 0.86) |
|  | | |  | | |
| **Sensitivity of the third trimester models based on EFW** ^d^ | | | **Sensitivity of the third trimester models based on EFW and placenta measures** ^d^ | | |
| **Specificity** | **LGA if gestational age adjusted birthweight >p95** | | **Specificity** | **LGA if gestational age adjusted birthweight >p95** | |
| 70% | 72% | | 70% | 67% | |
| 80% | 63% | | 80% | 61% | |
| 90% | 48% | | 90% | 43% | |

^a^ Receiver operating characteristic curves and corresponding area under the curve (95% confidence interval) of models based on predicted values from the five models for small- and large-size for gestational age. Small-size for gestational age defined as gestational age adjusted birth weight <5^th^ percentile. Large-size for gestational age defined as gestational age adjusted birth weight >95^th^ percentile.
^b^ Model 1: Maternal characteristics; Model 2: Maternal characteristics and 2^nd^ trimester EFW; Model 3: Maternal characteristics and 3^rd^ trimester EFW; Model 4: Maternal characteristics, 2^nd^ +3^rd^ trimester EFW; Model 5: Maternal characteristics, 2^nd^ + 3^rd^ trimester EFW and 2^nd^ to 3^rd^ trimester EFW change.
^c^ AUCs based on a sample of 2705 participants with all placenta measures available. Models as EFW models under ^b^, adding the following placenta measures. Model 2: 2^nd^ trimester uterine artery resistance index, and 2^nd^ trimester umbilical artery pulsatility index. Model 3; 3^rd^ trimester uterine artery resistance index, and 3^rd^ trimester umbilical artery pulsatility index; Model 4: 2^nd^ and 3^rd^  trimester uterine artery resistance index, and 2^nd^ and 3^rd^ trimester umbilical artery pulsatility index; Model 5: 2^nd^ and 3^rd^  trimester uterine artery resistance index, and 2^nd^ and 3^rd^ trimester umbilical artery pulsatility index, 2^nd^ to 3^rd^ trimester change of uterine artery resistance index and umbilical artery pulsatility index.
^d^ Effects of changing the outcome threshold on the sensitivity of the third trimester model used for screening of fetuses at risk for small-size and large-size for gestational age.

**Figure S9. Receiver operating characteristic curves for models screening for small-size and large-size for gestational age at birth defined as birth weight <3^rd^ and >97^th^ percentile, respectively^a^**

| **Small-size for gestational age as gestational age adjusted birthweight <3^rd^ percentile** | | | |
| --- | --- | --- | --- |
| **Estimated fetal weight^b^** | | **Estimated fetal weight and placenta measures^c^** | |
| 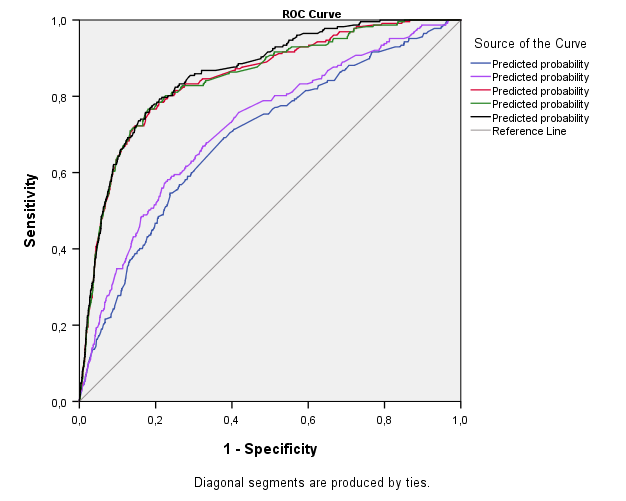 | Model 1: 0.69 (0.66 to 0.73)  (blue)  Model 2: 0.72 (0.69 to 0.76)  (purple)  Model 3: 0.85 (0.82 to 0.87)  (red)  Model 4: 0.85 (0.82 to 0.87)  (green)  Model 5: 0.86 (0.84 to 0.88)  (black) | 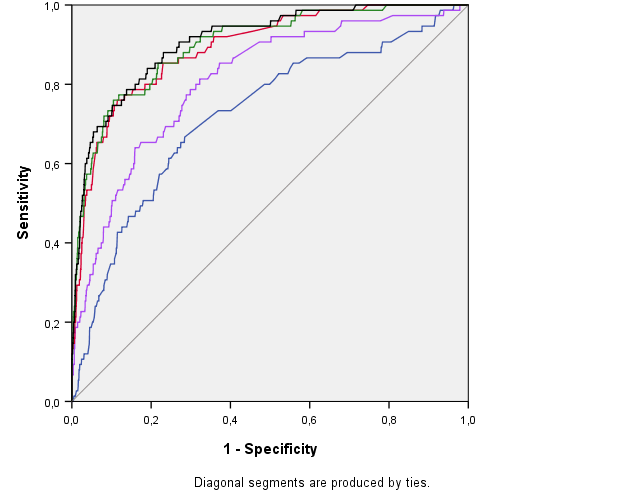 | Model 1: 0.72 (0.66 to 0.79)  (blue)  Model 2: 0.81 (0.76 to 0.86  (purple)  Model 3: 0.89 (0.76 to 0.93)  (red)  Model 4: 0.90 (0.86 to 0.94)  (green)  Model 5: 0.91 (0.88 to 0.94)  (black) |
|  |  |  |  |
| **Sensitivity of the third trimester models based on EFW** | | **Sensitivity of the third trimester models based on EFW and placenta measures** | |
| **Specificity** |  | **Specificity** |  |
| 70% | 83% | 70% | 86% |
| 80% | 77% | 80% | 80% |
| 90% | 64% | 90% | 68% |
|  |  |  |  |
| **Large-size for gestational age gestational age adjusted >97^th^ percentile** | | | |
| **Estimated fetal weight^b^** | | **Estimated fetal weight and placenta measures^c^** | |
| 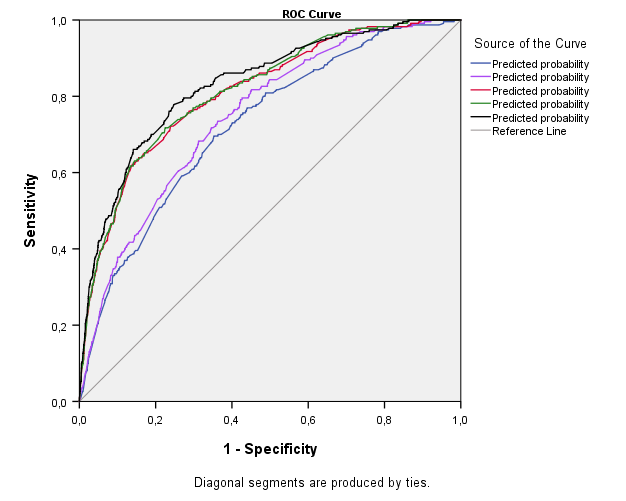 | Model 1: 0.72 (0.69 to 0.76)  (blue)  Model 2: 0.75 (0.72 to 0.78)  (purple)  Model 3: 0.81 (0.78 to 0.84)  (red)  Model 4: 0.82 (0.79 to 0.84)  (green)  Model 5: 0.83 (0.80 to 0.86)  (black) | 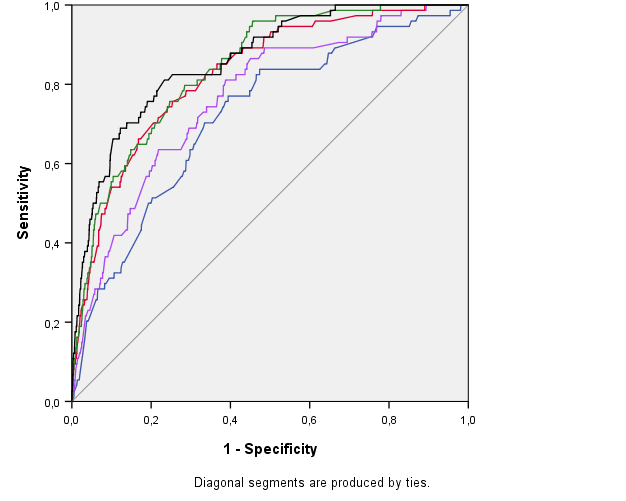 | Model 1: 0.72 (0.66 to 0.78)  (blue)  Model 2: 0.77 (0.71 to 0.82)  (purple)  Model 3: 0.83 (0.78 to 0.87)  (red)  Model 4: 0.84 (0.80 to 0.88)  (green)  Model 5: 0.86 (0.82 to 0.90)  (black) |
|  |  |  |  |
| **Sensitivity of the third trimester models based on EFW** | | **Sensitivity of the third trimester models based on EFW and placenta measures** | |
| **Specificity** |  | **Specificity** |  |
| 70% | 77% | 70% | 77% |
| 80% | 66% | 80% | 66% |
| 90% | 51% | 90% | 47% |

^a^ Receiver operating characteristic curves and corresponding area under the curve (95% CI) based on predicted values from five models for small- and large-size for gestational age. Models are based on maternal characteristics, second and third trimester fetal and placental ultrasound.
^b^ Model 1: Maternal characteristics; Model 2: Maternal characteristics and 2^nd^ trimester EFW; Model 3: Maternal characteristics and 3^rd^ trimester EFW; Model 4: Maternal characteristics, 2^nd^ +3^rd^ trimester EFW; Model 5: Maternal characteristics, 2^nd^ + 3^rd^ trimester EFW and 2^nd^ to 3^rd^ trimester EFW change.
^c^  AUCs based on a sample of 2705 participants with all placenta measures available. Models as EFW models under ^b^, adding the following placenta measures. Model 2: 2^nd^ trimester uterine artery resistance index, and 2^nd^ trimester umbilical artery pulsatility index. Model 3; 3^rd^ trimester uterine artery resistance index, and 3^rd^ trimester umbilical artery pulsatility index; Model 4: 2^nd^ and 3^rd^  trimester uterine artery resistance index, and 2^nd^ and 3^rd^ trimester umbilical artery pulsatility index; Model 5: 2^nd^ and 3^rd^  trimester uterine artery resistance index, and 2^nd^ and 3^rd^ trimester umbilical artery pulsatility index, 2^nd^ to 3^rd^ trimester change of uterine artery resistance index and umbilical artery pulsatility index.

**Figure S10. Receiver operating characteristic curves for models screening for small-size and large-size for gestational age at birth with adverse outcomes^a^**

| **Small-size for gestational age and gestational hypertensive disorders** | | | |
| --- | --- | --- | --- |
| **Estimated fetal weight^b^** | | **Estimated fetal weight and placenta measures^c^** | |
| 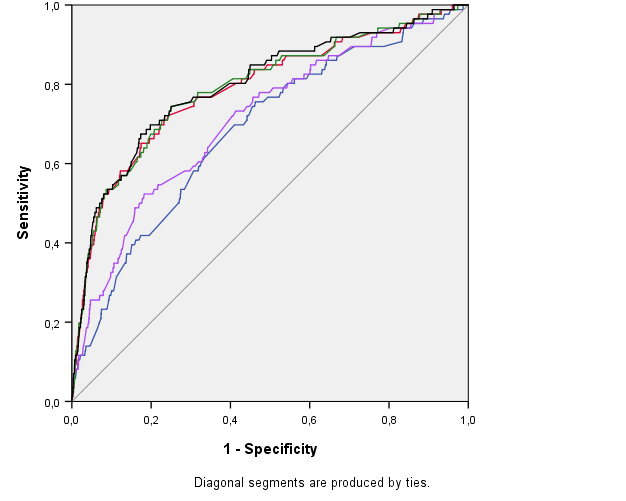 | Model 1: 0.68 (0.63 to 0.74)  (blue)  Model 2: 0.71 (0.66 to 0.77)  (purple)  Model 3: 0.79 (0.73 to 0.84)  (red)  Model 4: 0.79 (0.74 to 0.85)  (green)  Model 5: 0.80 (0.74 to 0.85) | 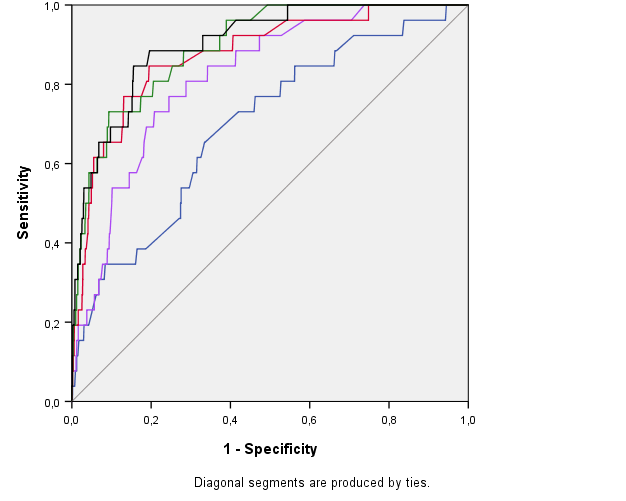 | Model 1: 0.70 (0.60 to 0.80)  (blue)  Model 2: 0.82 (0.75 to 0.89)  (purple)  Model 3: 0.88 (0.81 to 0.94)  (red)  Model 4: 0.90 (0.84 to 0.95)  (green)  Model 5: 0.90 (0.85 to 0.96)  (black) |
| **Large-size for gestational age and assisted vaginal delivery or cesarean section** | | | |
| **Estimated fetal weight^b^** | | **Estimated fetal weight and placenta measures^c^** | |
| 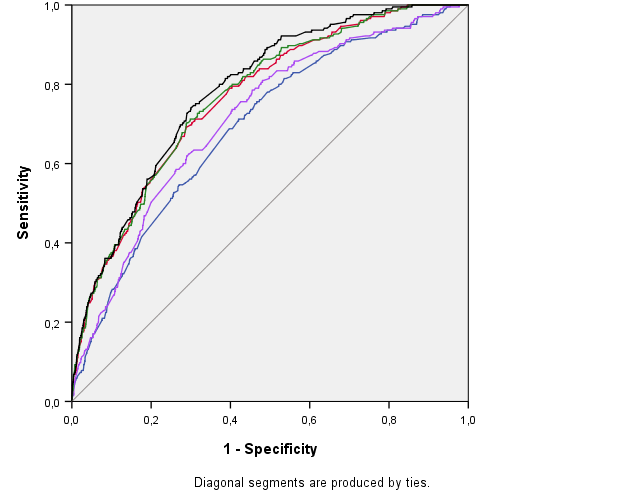 | Model 1: 0.69 (0.86 to 0.73)  (blue)  Model 2: 0.72 (0.68 to 0.75)  (purple)  Model 3: 0.77 (0.73 to 0.80)  (red)  Model 4: 0.77 (0.74 to 0.80)  (green)  Model 5:0.78 (0.76 to 0.81)  (black) | 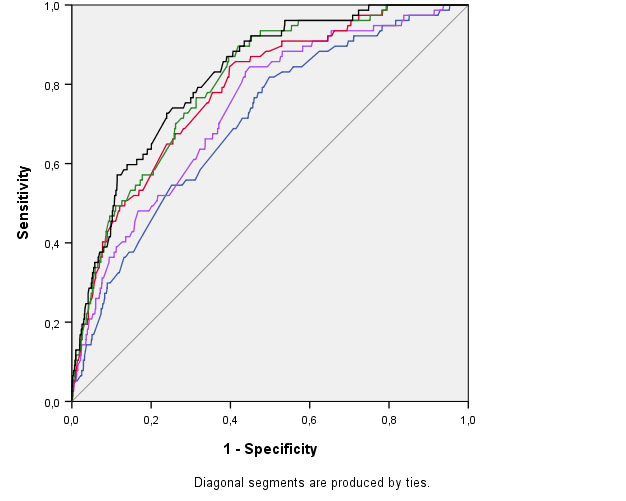 | Model 1: 0.70 (0.65 to 0.76)  (blue)  Model 2: 0.74 (0.68 to 0.79  (purple)  Model 3: 0.79 (0.74 to 0.83)  (red)  Model 4: 0.80 (0.76 to 0.85)  (green)  Model 5: 0.82 (0.78 to 0.86)  (black) |

^a^ Receiver operating characteristic curves and corresponding area under the curve (95% CI) based on predicted values from five models for small- and large-size for gestational age. Models are based on maternal characteristics, second and third trimester fetal and placental ultrasound.
^b^ Model 1: Maternal characteristics; Model 2: Maternal characteristics and 2^nd^ trimester EFW; Model 3: Maternal characteristics and 3^rd^ trimester EFW; Model 4: Maternal characteristics, 2^nd^ +3^rd^ trimester EFW; Model 5: Maternal characteristics, 2^nd^ + 3^rd^ trimester EFW and 2^nd^ to 3^rd^ trimester EFW change.
^c^ AUCs based on a sample of 2705 participants with all placenta measures available. Models as EFW models under ^b^, adding the following placenta measures. Model 2: 2^nd^ trimester uterine artery resistance index, and 2^nd^ trimester umbilical artery pulsatility index. Model 3; 3^rd^ trimester uterine artery resistance index, and 3^rd^ trimester umbilical artery pulsatility index; Model 4: 2^nd^ and 3^rd^  trimester uterine artery resistance index, and 2^nd^ and 3^rd^ trimester umbilical artery pulsatility index; Model 5: 2^nd^ and 3^rd^  trimester uterine artery resistance index, and 2^nd^ and 3^rd^ trimester umbilical artery pulsatility index, 2^nd^ to 3^rd^ trimester change of uterine artery resistance index and umbilical artery pulsatility index.
