# Supplementary material for: Second and third trimester fetal ultrasound population screening for risks of preterm birth and small-size and large-size for gestational age at birth: a population-based prospective cohort study: Fetal ultrasound screening for common adverse birth outcomes
Source: BMC Med. 2020 Apr 7;18:63. doi: 10.1186/s12916-020-01540-x (PMC7137302; doi:10.1186/s12916-020-01540-x)
Supplement: Supplementary file 2 — STROBE checklist. [file 12916_2020_1540_MOESM2_ESM.doc]

STROBE Statement—Checklist of items that should be included in reports of ***cohort studies***

|  | Item No | Recommendation |
| --- | --- | --- |
| **Title and abstract** | 1 | (*a*) Indicate the study’s design with a commonly used term in the title or the abstract  *In abstract, under subheading “Methods”, page 5* |
|  |  | (*b*) Provide in the abstract an informative and balanced summary of what was done and what was found  *Under “Abstract”, Page 2 and 3.* |
| Introduction | | |
| Background/rationale | 2 | Explain the scientific background and rationale for the investigation being reported  *Under “Background”, Page 4* |
| Objectives | 3 | State specific objectives, including any prespecified hypotheses  *Under “Background”, Page 4* |
| Methods | | |
| Study design | 4 | Present key elements of study design early in the paper  *Under “Subjects and methods”, subheading “study design”, Page 5* |
| Setting | 5 | Describe the setting, locations, and relevant dates, including periods of recruitment, exposure, follow-up, and data collection  *Under “Study design”, “Maternal characteristics at the start of pregnancy”, “Second and third trimester fetal and placental ultrasound”, and “Birth outcomes”, Page 5-7* |
| Participants | 6 | (*a*) Give the eligibility criteria, and the sources and methods of selection of participants. Describe methods of follow-up  *Eligibility criteria: Under “Study design”, page 6*  *Methods of follow up: Under “Study design”, “Maternal characteristics at the start of pregnancy”, “Second and third trimester fetal and placental ultrasound”, and “Birth outcomes”, Page 5-7* |
|  |  | (*b*) For matched studies, give matching criteria and number of exposed and unexposed  *Not applicable* |
| Variables | 7 | Clearly define all outcomes, exposures, predictors, potential confounders, and effect modifiers. Give diagnostic criteria, if applicable  *Under “Maternal characteristics at the start of pregnancy”, “Second and third trimester fetal and placental ultrasound”, and “Birth outcomes”, Page 5-7* |
| Data sources/ measurement | 8* | For each variable of interest, give sources of data and details of methods of assessment (measurement). Describe comparability of assessment methods if there is more than one group  *Under “Maternal characteristics at the start of pregnancy”, “Second and third trimester fetal and placental ultrasound”, and “Birth outcomes”, Page 6-8* |
| Bias | 9 | Describe any efforts to address potential sources of bias |
| Study size | 10 | Explain how the study size was arrived at  *Additional file 1, Figure S1* |
| Quantitative variables | 11 | Explain how quantitative variables were handled in the analyses. If applicable, describe which groupings were chosen and why  *Under “Maternal characteristics at the start of pregnancy”, page 5* |
| Statistical methods | 12 | (*a*) Describe all statistical methods, including those used to control for confounding  *Under”Statistical analyses”, page 7* |
|  |  | (*b*) Describe any methods used to examine subgroups and interactions  *Under”Statistical analyses”, page 7* |
|  |  | (*c*) Explain how missing data were addressed  *Under”Statistical analyses”, page 7* |
|  |  | (*d*) If applicable, explain how loss to follow-up was addressed  *Not applicable* |
|  |  | (*e*) Describe any sensitivity analyses  *Under”Statistical analyses”, page 7* |
| Results | | |
| Participants | 13* | (a) Report numbers of individuals at each stage of study—eg numbers potentially eligible, examined for eligibility, confirmed eligible, included in the study, completing follow-up, and analysed  *Additional file 1, Figure S1* |
|  |  | (b) Give reasons for non-participation at each stage |
|  |  | (c) Consider use of a flow diagram *Additional file 1, Figure S1* |
| Descriptive data | 14* | (a) Give characteristics of study participants (eg demographic, clinical, social) and information on exposures and potential confounders  *Table 1* |
|  |  | (b) Indicate number of participants with missing data for each variable of interest *Supplemental figure 1, supplement page 2* |
|  |  | (c) Summarise follow-up time (eg, average and total amount)  *Under “Study design”, page 5* |
| Outcome data | 15* | Report numbers of outcome events or summary measures over time  *Table 2, page 24* |
| Main results | 16 | (*a*) Give unadjusted estimates and, if applicable, confounder-adjusted estimates and their precision (eg, 95% confidence interval). Make clear which confounders were adjusted for and why they were included  *Figure 1,2,3* |
|  |  | (*b*) Report category boundaries when continuous variables were categorized  *Under “Maternal characteristics at the start of pregnancy”, “Second and third trimester fetal and placental ultrasound”, and “Birth outcomes”, Page 5-8* |
|  |  | (*c*) If relevant, consider translating estimates of relative risk into absolute risk for a meaningful time period |
| Other analyses | 17 | Report other analyses done—eg analyses of subgroups and interactions, and sensitivity analyses  *Supplemental figures S3,S4,S5,S6* |
| Discussion | | |
| Key results | 18 | Summarise key results with reference to study objectives  *Under “Discussion”, page 14* |
| Limitations | 19 | Discuss limitations of the study, taking into account sources of potential bias or imprecision. Discuss both direction and magnitude of any potential bias  *Under “Strengths and limitations”, page 17* |
| Interpretation | 20 | Give a cautious overall interpretation of results considering objectives, limitations, multiplicity of analyses, results from similar studies, and other relevant evidence  *Under “Interpretation of main findings”, page 14-17* |
| Generalisability | 21 | Discuss the generalisability (external validity) of the study results  *Under “Strengths and limitations”, page 17* |
| Other information | | |
| Funding | 22 | Give the source of funding and the role of the funders for the present study and, if applicable, for the original study on which the present article is based  *Under “Funding”, page 18* |

*Give information separately for exposed and unexposed groups.
